# Integrated single-cell and spatial transcriptomics reveal stromal–immune–vascular crosstalk in patients with interstitial cystitis/bladder pain syndrome

**DOI:** 10.1038/s12276-026-01768-2

**Published:** 2026-07-03

**Authors:** ByulA Jee, IJun Kim, Cheol Lee, Yeon Jeong Kim, Kyunghee Park, Ji-Hee Sung, Inwoo Hwang, Jung-Sun Kim, Kyu-Sung Lee, Kwang Jin Ko, Minyong Kang

**Affiliations:** 1 Department of Urology, Samsung Medical Center, Sungkyunkwan University School of Medicine, Seoul, South Korea; 2 Department of Health Sciences and Technology, SAIHST, Sungkyunkwan University, Seoul, South Korea; 3 Department of Pathology, Seoul National University Hospital, Seoul National University College of Medicine, Seoul, South Korea; 4 Translational Genomics Center, Samsung Medical Center, Seoul, South Korea; 5 Department of Obstetrics and Gynecology, Samsung Medical Center, Sungkyunkwan University School of Medicine, Seoul, South Korea; 6 Department of Pathology and Translational Genomics, Samsung Medical Center, Sungkyunkwan University School of Medicine, Seoul, South Korea

**Keywords:** Translational research, Urinary incontinence, Genome informatics

## Abstract

Interstitial cystitis/bladder pain syndrome (IC/BPS) is characterized by complex cellular heterogeneity and inflammatory dysfunction in the bladder. However, the molecular pathogenesis of IC/BPS remains poorly understood, and few studies have provided comprehensive analyses. To elucidate the mechanisms underlying disease pathology, we performed integrated single-cell and spatial transcriptomic analyses of human bladder tissue. Comprehensive single-cell multiomics analyses have identified disease-specific alterations across multiple cell types. T helper 17 cells were significantly expanded in Hunner lesions and activated autoimmune and pro-inflammatory signaling pathways, suggesting a critical role in disease progression. Endothelial cells (ECs) shift from homeostatic (EC2) to pro-angiogenic inflammatory phenotypes (EC3), contributing to vascular remodeling. In the monocyte–macrophage lineage, we identified macrophage 2 cells with pro-angiogenic features that engaged in vascular endothelial growth factor-mediated and tumor necrosis factor-mediated interactions that promoted pathological angiogenesis. Smooth muscle cells (SMCs) display notable phenotypic plasticity, transitioning from a contractile to a synthetic state in IC/BPS tissues. Additionally, spatial transcriptomic analysis revealed upregulation of the EDN1–EDNRA/EDNRB signaling axis, suggesting a role for SMCs in bladder hypercontractility. Fibroblasts have emerged as key mediators of nociceptive signaling, with specific subpopulations enriched in neurotrophin-related pathways (for example, NTF3–NTRK2/NTRK3 and NGF–SORT1) and showing active crosstalk with SMCs, potentially contributing to the development of chronic pain. Collectively, these findings delineate a complex stromal–immune–vascular network underlying the pathological remodeling and pain characteristics of IC/BPS. Our study identified novel cellular players and intercellular signaling pathways that may be promising targets for therapeutic interventions.

## Bladder pain syndrome revealed through single-cell analysis

Interstitial cystitis/bladder pain syndrome is a chronic urological condition marked by pelvic pain and urinary issues without clear infection. Despite its impact, its pathophysiology is not well understood, and treatments are inconsistent. This study focusses on Hunner-type IC, a distinct subtype with inflammatory lesions, using single-cell and spatial transcriptomic analyses to explore cellular and molecular features of bladder tissues. Researchers compared Hunner lesions and non-Hunner lesions to identify specific cellular differences. They found significant variations in immune, stromal, and vascular cells, highlighting the role of T helper 17 cells in inflammation and pain. The study also noted endothelial and smooth muscle cell changes, suggesting potential therapeutic targets such as EDN1 signaling. These findings offer insights into interstitial cystitis/bladder pain syndrome pathogenesis and point to future research directions, including exploring immune modulation and pain management strategies.

This summary was initially drafted using artificial intelligence, then revised and fact-checked by the author.

## Introduction

Interstitial cystitis/bladder pain syndrome (IC/BPS) is a chronic and debilitating urological condition characterized by pelvic pain, urinary urgency, and frequency in the absence of identifiable infection or overt pathological causes^1–3^. Despite its substantial impact on the quality of life and clinical management, the pathophysiology of IC/BPS remains incompletely understood, and therapeutic options are often ineffective or inconsistent^2,4,5^.

Among the various clinical subtypes, Hunner-type IC, defined by the presence of discrete inflammatory lesions observed on cystoscopy, has emerged as a biologically distinct and clinically meaningful entity^6–9^. These Hunner lesions (HLs) exhibit characteristic pathological features, including urothelial denudation, dense lymphoplasmacytic infiltration, stromal fibrosis, and angiogenesis, indicating a highly active and localized inflammatory process^9^. As a result, Hunner-type IC is increasingly regarded as a distinct disease within the broader IC/BPS spectrum rather than a simple variant.

Interestingly, cystoscopic observation alone may not reflect the full extent of bladder pathology in patients with Hunner-type IC. Even areas of the bladder that appear endoscopically “normal” or non-Hunner lesions (NHLs) often demonstrate histological evidence of chronic inflammation and tissue remodeling, raising the possibility that HLs represent focal exacerbations of a more widespread pathological process. However, whether these visual NHLs are biologically distinct from HLs or represent transitional states in the progression toward lesion formation remains an open and clinically relevant question.

Single-cell RNA sequencing (scRNA-seq) has revolutionized our ability to dissect complex tissues by enabling transcriptomic profiling of individual cells. This technology provides unprecedented insights into cellular diversity, lineage relationships, and transcriptional states associated with the disease^10,11^. However, most previous single-cell studies in patients with IC/BPS did not differentiate between HLs and NHLs within the same bladder, potentially overlooking important spatial and pathological distinctions^12,13^. Furthermore, key pathophysiological processes such as autoimmunity, angiogenesis, bladder contractility, and pain mechanisms have rarely been explored in previous studies^12,13^.

To address these critical gaps, we performed integrative single-cell and spatial transcriptomic analyses of bladder tissue from patients with Hunner-type IC and healthy controls. We macroscopically analyzed the tissues from both HLs and NHLs in each IC bladder. By comparing HLs and NHLs within the same bladder, we aimed to identify lesion-specific cellular and molecular features while minimizing interpatient variability. This approach enabled the investigation of whether NHLs represent a prelesional state, thereby providing insights into the spatial dynamics and potential progression of the disease in Hunner-type IC.

Our findings revealed spatially organized differences in the immune, stromal, and vascular cell populations and their molecular programs, offering novel insights into the pathophysiology of Hunner-type IC and uncovering potential targets for early intervention and disease-modifying therapies.

## Materials and methods

### Sample participants and tissue collection

Patients aged more than 20 years diagnosed with IC/BPS and reported bladder pain with a visual analog score (VAS) of 4 or higher persisting for more than 6 months were screened for eligibility. Only patients with cystoscopically confirmed HLs were included in this study. Among these, eight patients who had either undergone multiple prior endoscopic ablation treatments or had reached a state in which endoscopic intervention was no longer feasible and therefore elected to undergo supratrigonal cystectomy with augmentation cystoplasty were enrolled.

Full-thickness bladder tissue samples were obtained during the surgery. Samples were exclusively collected from HLs in three of the eight patients. In the remaining five patients, tissue samples were obtained from both the HLs and areas that appeared grossly normal and were referred to as NHLs. Control bladder tissue samples were obtained from two individuals who underwent pelvic surgery for benign conditions. One control sample was collected during robot-assisted simple prostatectomy for benign prostatic hyperplasia, and the other was acquired during ureteroneocystostomy with a psoas hitch for ureteral stricture. In both cases, bladder tissue was obtained from areas unrelated to the primary surgical site.

For patients with IC/BPS, clinical data, including a 3-day voiding diary, interstitial cystitis symptom index, interstitial cystitis problem index, pelvic pain and urgency/frequency symptoms, bother scores, and VAS for pain, were obtained preoperatively.

In parallel to the aforementioned sample acquisition process, whole transcriptome sequencing data were collected from 35 patients with IC/PBS at the Samsung Medical Center. Whole transcriptome sequencing samples were prospectively obtained from 2021 to 2022, whereas samples from 10 patients, including 2 normal and 8 IC/BPS samples, were prospectively collected between July 2023 and January 2024 for single-cell and spatial transcriptomic analyses.

The Institutional Review Board of our center approved the use of human archival or fresh tissues obtained from patients in this study. Written informed consent was obtained from all enrolled patients who survived during the study period (IRB nos. SMC-2023-04-112 and 2021-10-086). Any personal identifiers were removed using an anonymized processing protocol, and all methods used in this study were performed in accordance with the Declaration of Helsinki.

### RNA-seq preprocess

To perform RNA-seq using 7 samples with normal tissues, 18 samples with NHLs, and 17 samples with HLs, total RNA was extracted using an RNA extraction kit (RNeasy Mini Kit, QIAGEN, MD, USA), and RNA integrity was verified using a 2100 Bioanalyzer (Agilent, Snata Clara, CA, USA). Libraries for sequencing were generated using the QuantSeq 3ʹ Library Prep Kit (Lexogen Inc., Vienna, Austria) according to the manufacturer’s instructions and sequenced on a HiSeq 2000 system (Illumina, San Diego, CA, USA). The reads were mapped to the GRCh38 human reference genome using STAR (version 2.5.2b) with default parameters. The number of reads mapped to each gene was calculated using RNA-seq with RSEM (version 1.3.0). Genes with zero values across samples were filtered out to preprocess the transcriptome data. Data processing and analysis were performed using the R/Bioconductor libraries.

### scRNA-seq and data analysis

To perform scRNA-seq using 15 tissues, including Hunner, non-Hunner, and normal tissues, 10× Chromium Chip single-cell library generation and preparation were performed on the single-cell suspension according to the Chromium Single Cell 3' Reagent v2 kit (10x Genomics, Pleasanton, CA, USA) as per the manufacturer’s instructions with the aim to capture 5,000–10,000 cells per channel. Sequencing was performed by Geninus Inc (Seoul, South Korea). using an Illumina HiSeq 4000 platform. Raw sequencing data were processed using Cell Ranger software (version 3.0.2, 10× Genomics) and aligned to the human reference genome (GRCh38). Low-quality cells and potential doublets were excluded from downstream analyses based on the following criteria: cells expressing fewer than 500 or more than 8000 genes or exhibiting more than 15% mitochondrial gene expression. Additionally, doublets were identified and removed using the scDblFinder package (version 1.22.0)^14^. For the remaining 102,384 cells, gene expression data were normalized using the NormalizeData function and scaled using the ScaleData function from the Seurat package (version 4.4.0). Batch effects were corrected using the RunHarmony function with sample identity specified as batch variable (Supplementary Fig. 2a) and implemented using the Seurat package^15^.

### Regions of interest selection, spatial transcriptomic sequencing, and data analysis

Two pathologists (C.L. and I.H.) independently reviewed regions of interest in bladder tissue sections while blinded to the clinical data. Spatial transcriptomic data were generated using the Visium Spatial Gene Expression platform (10× Genomics) according to the manufacturer’s instructions. This platform captures mRNA using spatially barcoded oligonucleotides, enabling spatially resolved gene expression profiling. Raw sequencing reads were quality checked and aligned to the human reference transcriptome (GRCh38) using Space Ranger (version 2.0.1). The resulting gene spot count matrices were processed in R using the Seurat package (version 4.4.0) for normalization, feature selection, and dimension reduction. Spots with mitochondrial transcript proportions exceeding 15% were excluded from further analysis.

To infer the spatial distribution of cell types across tissue sections, we applied the conditional autoregression-based deconvolution method. This method integrates matched scRNA-seq data to estimate the cell-type proportions for each spatial spot while accounting for spatial autocorrelation and platform-specific variability. The inferred cell-type proportions were added to the Seurat object metadata, and the dominant cell type for each spot was assigned based on the highest estimated proportion. This annotation enabled cell-type-specific spatial visualization and guided the downstream functional analyses.

### Visium HD data generation

To validate the results derived from Visium transcriptomic data, spatial transcriptomics libraries were generated from six formalin-fixed paraffin-embedded specimens (three normal and three IC/BPS samples) using the 10× Genomics Visium HD platform. Tissue sections (5 μm) were mounted on Visium HD CytAssist slides, followed by hematoxylin and eosin (H&E) staining and imaging. RNA quality was assessed using DV200 analysis. Following probe hybridization and ligation with the Human Transcriptome Probe Kit v2.0, transcripts were enzymatically transferred to 2 μm barcoded bins using the 10× Genomics CytAssist instrument. Libraries were sequenced on an Illumina NextSeq 2000 to achieve high-resolution spatial transcriptomic profiling.

### Visium HD data processing and cell segmentation

Visium HD data from six specimens were processed using Space Ranger (version 4.0.1) with the GRCh38 reference and corresponding H&E images to generate gene-count matrices. To achieve near single-cell resolution, cell segmentation was performed based on H&E-derived nuclear boundaries. Transcripts were mapped to segmentation masks (polygons), and only transcripts contained within each boundary were aggregated to generate the final segmented count matrix. Polygons with fewer than 200 unique molecular identifiers or genes, or with more than 25% mitochondrial gene expression, were excluded from downstream analysis.

### Spatial domain identification

Spatial domains were identified using BANKSY (version 1.6.0), which integrates individual transcriptomic profiles with spatial neighborhood information. The spatial weighting parameter (lambda) was set to 0.2 to balance local gene expression and regional context, enabling the identification of coherent tissue niches. This approach facilitated refined characterization of tissue architecture and microenvironmental organization for downstream interaction analysis.

### Cell-type deconvolution of spatial transcriptomics data

Cell-type composition within each spatial location was inferred using spacexr (version 2.2.1). Deconvolution was performed using the robust cell-type decomposition algorithm in doublet mode, which assigns up to two cell types per spot and is well suited for high-resolution spatial transcriptomics platforms such as Visium HD.

### Find marker genes in each cluster

Differentially expressed marker genes for each cluster were identified using the FindAllMarkers function of the Seurat package with the default settings. Genes were considered significant if they exhibited adjusted *P*-values less than 0.05 and an average log_2_ fold-change greater than 1.0.

### Pseudotime trajectory analysis

Trajectory analysis was performed using Monocle2 (version 2.30.1) to elucidate the developmental pathways of macrophages, monocytes, and smooth muscle cells (SMCs)^16^. Cells from each lineage were independently analyzed to construct pseudotime trajectories and explore lineage progression and transitional states. Dimensionality reduction was conducted using the DDRTree algorithm, and the resulting embeddings were used for trajectory visualization. Following cell ordering, the trajectories were visualized using the plot_cell_trajectory function.

To further investigate the gene expression dynamics at branch points, the Branch Expression Analysis Modeling function in Monocle2 was applied. This enabled the identification of branch-dependent gene expression changes, which were visualized using branched heat maps. In addition, to infer cell lineages and developmental trajectories from the endothelial cells (ECs), we used the Slingshot R package (version 2.12.0) utilizing the getLineage function^17^.

### GSEA and ssGSEA

To perform gene set enrichment analysis (GSEA), the hallmark gene sets from the Molecular Signatures Database (MSigDB version 7.0) were used^18^. Additionally, gene sets related to autoimmunity, cytokines, growth factors, and contraction were downloaded from the MsigDB database, which includes resources such as Gene Ontology (GO), Kyoto Encyclopedia of Genes and Genomes (KEGG), and REACTOME. Single-sample GSEA (ssGSEA) was computed using the GSVA package (version 2.4.8)^19^. ssGSEA for scRNA-seq was computed using the escape package (version 2.4.0). Signature scoring derived from spatial transcriptomic signatures was performed using the AddModuleScore function with the default parameters in Seurat. Spatial feature expression plots were generated using the spatial feature plot function in Seurat (version 4.4.0). The following specific gene signatures were analyzed^20,21^: contractile SMCs: *MYH11*, *ACTA2*, *TAGLN*, *CNN1*, *TPM1*, *TPM2*, *MYL9*, *LMOD1*, *DES*, *MYLK*, *PPP1R14A*, *ACTC1*, and *ACTG2*; synthetic SMCs: *THBS1*, *TNC*, *ELN*, *BGN*, *ADAMTS1*, *CTNNB1*, *SERTAD1*, *ITGA8*, *ZFHX3*, *KLF4*, and *KLF2*; inflammatory SMCs: *TIMP3*, *TIMP1*, *TIMP2*, *TCF21*, *SOX9*, *CD36*, *MMP2*, *MMP9*, *APOE*, *LOX*, *RUNX2*, *PDGFRB*, *SPP1*, *PCNA*, *POSTN*, *IL6*, *IL1B*, *CCL2*, *CXCL1*, *CXCL8*, *CXCL2*, *IRF1*, *FOS*, *JUN*, and *KLF4*.

### Regulon analysis

To infer gene regulatory networks and assess transcription factor (TF) activity in T helper 17 (T_H_17) cells across different tissue types, we applied Single-Cell Regulatory Network Inference and Clustering using the SCENIC R package (version 1.1.2) with the human hg38 transcriptional regulator database^22^. GENIE3 was used to identify candidate target genes that were co-expressed with TFs. Regulons were defined by integrating co-expression results with DNA motif enrichment analyses. To quantify regulon activity across tissue types, area under the curve (AUC) scores were computed using the AUCell function.

### Cell–cell interaction

Cell–cell interaction analysis was performed using CellphoneDB (version 5.0.0), a Python-based computational tool for inferring molecular interactions between cell types^23^. The analysis was conducted in the statistical analysis mode using normalized gene expression matrices and corresponding cell-type annotations as input. Ligand–receptor pairs were considered only if both components were expressed in at least 10% of the cells within a given cluster. The results were visualized using dot plots to identify the enriched signaling interactions between specific cell types.

### Cell-type proportion and three SMC subtypes

The proportions of T cell and EC subclusters were estimated from external validation datasets using CIBERSORTx^24^. Gene signatures consisting of differentially expressed genes (DEGs) specific to each subcluster were used as inputs, enabling CIBERSORTx to deconvolute the bulk transcriptomic profiles and estimate the relative abundance of each subpopulation. For SMC subtypes, including contractile, inflammatory-like, and synthetic SMCs, the nearest template prediction algorithm was applied based on subtype-specific gene expression signatures to classify the SMC phenotypes.

### Statistical analysis

GO analysis of DEGs was performed using the DAVID website (https://david.ncifcrf.gov) and the clusterProfiler (version 4.18.4) R package. Student’s *t* test was performed for both groups. All statistical analyses were performed using R software.

### Validation sets

To validate our findings, datasets were obtained from GEO (accession numbers: GSE238208, GSE57560, and GSE11783). For example, GSE238208 includes data from 25 HL and 25 NHL samples. GSE57560 comprised 3 normal samples and 13 IC/BPS samples, whereas GSE11783 contained 6 normal and 10 IC/BPS samples. Additionally, an in-house dataset was generated consisting of 7 normal, 18 NHL, and 17 HL samples. The external validation dataset included 16 normal and 108 IC/BPS samples (43 NHL and 42 HL samples). scRNA-seq data were obtained from GSE175526, comprising 2 controls and 5 IC/BPS samples, and from GSE310557, including 7 controls and 10 IC/BPS samples. An external scRNA-seq dataset consisted of 9 controls and 15 IC/BPS samples. In addition, we generated high-resolution spatial transcriptomics data using the 10× Visium HD platform from an independent cohort of 3 controls and 3 patients with IC/BPS. These Visium HD datasets were used to specifically assess disease-associated ligand–receptor interactions identified in the scRNA-seq and Visium analysis.

## Results

### Clinical characteristics of patients with IC/BPS

Detailed clinical information of the study participants is provided in Supplementary Tables 1 and 2. This includes the baseline characteristics of the eight patients with Hunner-type IC/BPS: the median age was 64.0 years (range: 55.5–72.6), and 62.5% were male. The median number of previous endoscopic ablations was 3.0 (range: 2–5). The median maximal cystometric capacity was 195.0 ml (range: 80.0–280.0 ml), with a mean bladder capacity of 122.5 ml (range: 57.0–178.0 ml). Patients reported a median urinary frequency of 15.4 episodes/day (range: 9.3–33.3). Symptom severity scores included a median O’Leary–Sant interstitial cystitis symptom index of 15.5 (range: 13.0–20.0) and problem index of 15.0 (range: 11.0–16.0). The median pelvic pain and urgency/frequency (PUF) symptom score was 17.0 (range: 10.0–21.0), and the bothersome score was 8.5 (range: 5.0–10.0). The median VAS pain score was 7.5 (range: 4.0–10.0).

### Overall distribution of single-cell transcriptomic profiles in IC/BPS

**Fig. 1:**
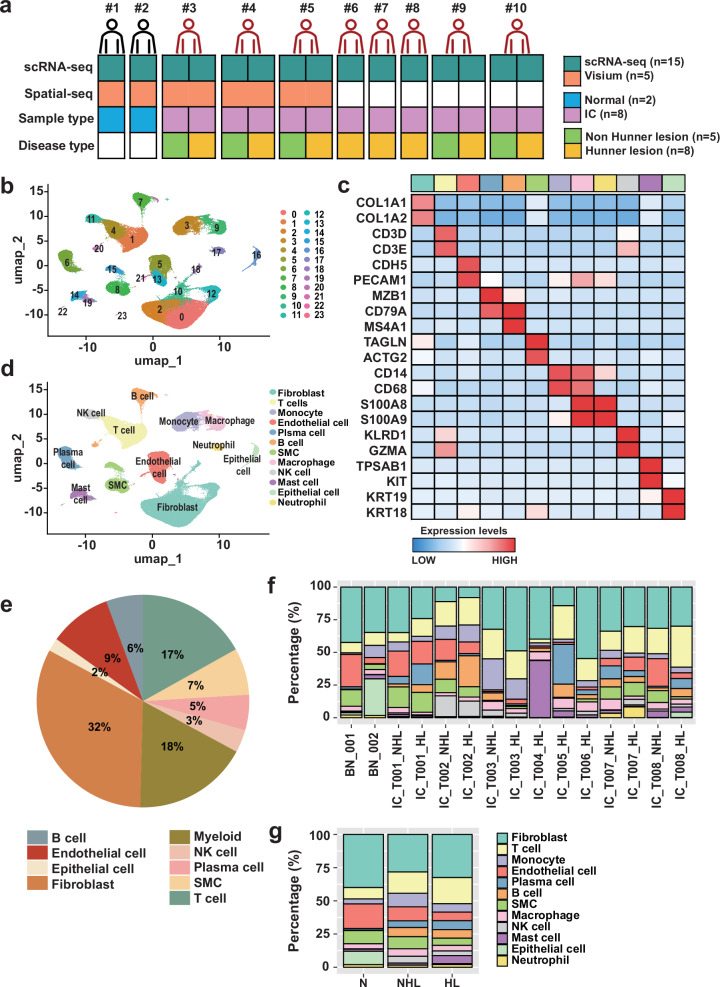
Overall distribution of single-cell transcriptomic profiles in IC/BPS. **a** Overview of sample composition used for single-cell RNA sequencing (scRNA-seq) and spatial transcriptomic (ST) analysis. scRNA-seq data were generated from 2 normal controls and 13 interstitial cystitis/bladder pain syndrome (IC/BPS) bladder tissues. ST was established based on 3 IC/BPS and 2 control bladders tissues. A total of 10 bladder tissue samples were obtained from 2 normal controls and 8 patients with IC/BPS, including regions classified as non-Hunner lesions and Hunner lesions. Each row indicates the data modality, sample type, and corresponding disease type. **b** UMAP plot showing clustering of cells into 24 transcriptionally distinct clusters. **c** Heatmap displaying the expression of representative marker genes across 12 major cell types. **d** UMAP plot showing the annotation of 12 major cell types. **e** Pie chart summarizing the proportion of nine major cell types (myeloid cells comprising monocytes, macrophages, neutrophils, and mast cells). Stacked bar plots showing the proportion of 12 major cell types across individual samples (part **f**) and aggregated by tissue types (part **g**). HL, Hunner lesion; N, normal; NHL, non-Hunner lesion; NK, natural killer; SMC, smooth muscle cell; UMAP, uniform manifold approximation and projection.

To comprehensively characterize the cellular landscape of IC/BPS, we conducted scRNA-seq and spatial transcriptomic analyses of surgically obtained bladder tissues from patients with IC/BPS (*n* = 8) and healthy controls (*n* = 2) (Fig. 1a and Supplementary Fig. 1a–c). In the IC/BPS group, paired samples were collected from both NHL and HL areas, followed by supratrigonal cystectomy with augmentation cystoplasty.

Unsupervised clustering of single-cell transcriptomes identified 24 distinct cellular clusters (Fig. 1b). Marker gene analysis enabled the annotation of major cell types, including fibroblasts (*COL1A1* and *COL1A2*), ECs (*CDH5* and *PECAM1*), epithelial cells (*KRT18* and *KRT19*), T cells (*CD3D* and *CD3E*), B cells (*CD79A* and *MZB1*), and myeloid cells (*CD68* and *CD14*) (Fig. 1c, d). Among all cell populations, fibroblasts (32%) and myeloid cells (18%) constituted the largest fractions in the dataset, followed by T cells and ECs (Fig. 1e). The relative proportions of each cell type varied considerably across the individual samples and lesion types. Notably, fibroblasts were the predominant cell population in most samples (Fig. 1f, g. and Supplementary Fig. 2b, c). Dynamic changes in the abundance of various cell types were observed depending on the tissue and disease context (Supplementary Fig. 2c). These findings underscore the cellular heterogeneity of IC/BPS and suggest that diverse cell populations, particularly fibroblasts and various immune cells, have distinct roles in its pathophysiology.`

**Fig. 2:**
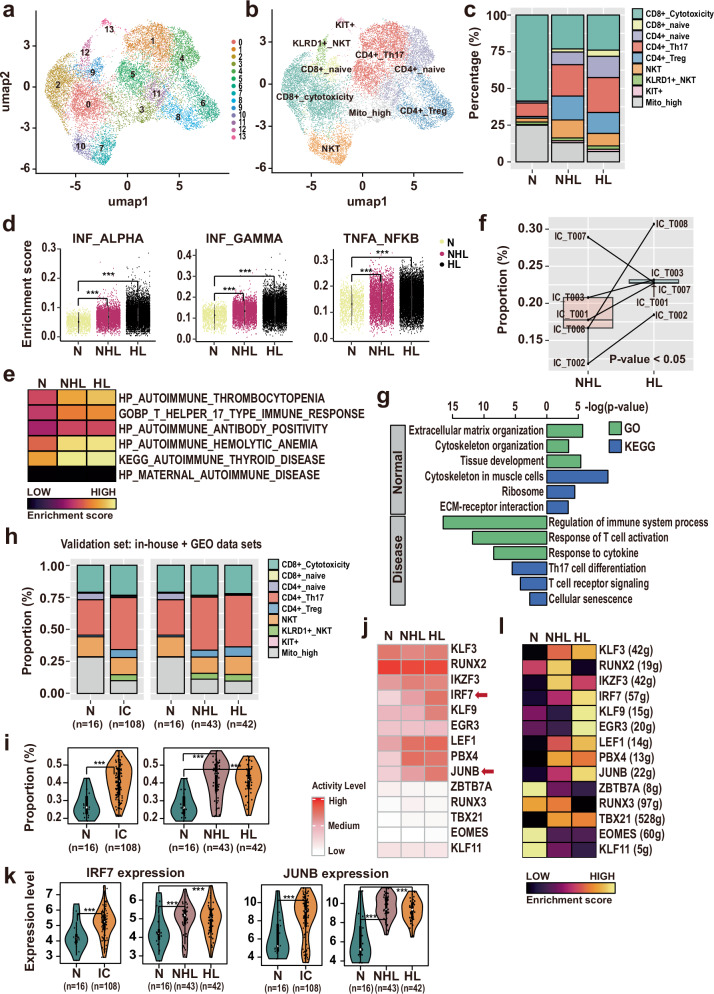
Functional characterization of T cell subclusters and validation using external validation datasets. **a** Uniform manifold approximation and projection (UMAP) plot showing 14 T cell subclusters. **b** UMAP plot displaying the annotation of the T cell subclusters. **c** Stacked bar plots showing the proportion of T cell subclusters aggregated by tissue type. **d** Box plots illustrating single-sample gene set enrichment analysis (ssGSEA) scores of interferon-α (INF_ALPHA), interferon-γ (INF_GAMMA), and tumor necrosis factor-alpha (TNFα) signaling via nuclear factor (NF)-κB (TNF_NF-κB) pathways across tissue types. **e** Heatmap of GSEA scores of gene sets associated with autoimmune diseases across tissue types. **f** Box plot showing the proportion of T helper 17 (T_H_17) subclusters using five paired samples, including NHL and HL (*P*-value was determined using Fisher’s exact test). **g** Gene Ontology (GO) and Kyoto Encyclopedia of Genes and Genomes (KEGG) pathway analyses using differentially expressed genes in T_H_17 cells between normal and diseased tissues (including NHL and HL). **h** Stacked bar plots showing the proportion of nine T cell subclusters in the external validation datasets estimated by CIBERSORTx. **i** Violin plots showing the proportion of T_H_17 cells in different tissue types. **j** Heatmap showing the activity of transcription factors (TFs) in different tissue types. **k** Violin plots showing the expression levels of *IRF7* and *JUNB* in the external validation datasets. **l** Heatmap of GSEA scores of gene sets associated with target genes of each TF across tissue types. The right panel lists TFs, with the number of their target genes indicated in parentheses (significance was determined using Student’s *t* test). NS, not significant; *P < 0.05; **P < 0.01; ***P < 0.001. ECM, extracellular matrix; HL, Hunner lesion; IC, interstitial cystitis; N, normal; NHL, non-Hunner lesion.

### Increase of T_H_17 cell type was associated with progression of IC/BPS

To investigate alterations in individual T cell subsets between the IC/BPS and normal groups, we performed re-clustering of T cells, resulting in the identification of nine distinct subclusters based on the expression of canonical T cell markers (Fig. 2a, b and Supplementary Fig. 3a). Among these, we identified a T_H_17 cell subcluster characterized by upregulation of the *CD40LG* gene, which encodes a membrane-bound cytokine involved in the activation and differentiation of T_H_17 cells^25^. Furthermore, the proportions of seven subclusters were increased in the IC/BPS group, except for two subclusters associated with CD8^+^ cytotoxicity and high mitochondrial gene expression, which were decreased (Fig. 2c and Supplementary Fig. 3b). Although the relative proportion of cytotoxicity CD8⁺ T cells was reduced in IC/BPS tissues, this decrease did not correspond to diminished activity at the transcriptional level. GSEA revealed progressively increased enrichment of activated T cell-related and cytotoxicity-related gene signatures^26^ from N (normal) to NHLs and further to HLs, indicating enhanced functional activation of the remaining T cell populations despite reduced cell abundance (Supplementary Fig. 3c).

To further elucidate the key biological pathways associated with IC/BPS progression, we conducted GSEA using 50 hallmark gene sets. In total, nine gene sets — primarily related to inflammatory response, interferon-α/γ response, and tumor necrosis factor (TNF) signaling— were significantly enriched in both NHLs and HLs, but not in N tissues (Fig. 2d and Supplementary Fig. 3d,e). These results consistently indicate that gene sets related to inflammatory responses are markedly activated in IC/BPS tissues^12,13^.

Previous studies have suggested that IC/BPS may be influenced by autoimmune mechanisms^27^. To further investigate this hypothesis, we analyzed six autoimmune-related gene sets obtained from the MSigDB database^18^. All gene sets, except for the one associated with maternal autoimmune disease, showed increased enrichment in IC/BPS tissues compared with normal tissues (Fig. 2e and Supplementary Fig. 3f,g). These findings support a potential autoimmune basis for IC/BPS. To assess the distribution of autoimmune-associated programs across T cell subsets, we compared enrichment scores among major T cell populations. Autoimmune-related gene signatures were detected across multiple immune cell types, including regulatory T cells and cytotoxic CD8⁺ T cells, consistent with a broadly activated inflammatory microenvironment in IC/BPS. Notably, T_H_17 cells displayed the strongest enrichment of autoimmune-associated gene signatures among T cell subsets (Supplementary Fig. 3f). Elevated scores were also observed in a small KIT^+^ T cell population, although this subset represented a minor fraction of total T cells.

Notably, the analysis of T cell subcluster proportions revealed a significant increase in T_H_17 cells in the IC/BPS tissues (Fig. 2c). Among the five paired samples comprising both NHLs and HLs, four exhibited a higher proportion of T_H_17 cells in HLs than in the matched NHLs, except for one sample (IC_T007) (Fig. 2f). To further investigate their functional relevance, GO and KEGG pathway enrichment analyses were performed on DEGs from T_H_17 cells in the IC/BPS group compared with those in the normal group. These DEGs were primarily enriched in pathways related to immune system regulation, T cell activation, T_H_17 cell differentiation, and cellular senescence (Fig. 2g). Similarly, GO and KEGG analyses of DEGs from T_H_17 cells in HLs compared with those in NHLs revealed enrichment in pathways involved in the regulation of apoptotic processes and TNF signaling (Supplementary Fig. 4a). These findings suggested that T_H_17 cells undergo senescence and contribute to the inflammatory response associated with IC/BPS^28^.

To validate these findings, we used external whole transcriptome datasets, including in-house and public data, to estimate the proportion of T cell types based on our defined T cell subclusters. The overall distribution of T cell subclusters was consistent with the results of our single-cell transcriptomic analysis (Fig. 2h and Supplementary Fig. 4b). Notably, the proportion of T_H_17 cells was significantly higher in the IC/BPS tissues than in the normal tissues (Fig. 2i). In addition, validation of the hallmark and autoimmunity-related gene sets showed results consistent with those obtained from the T cell subcluster analysis (Supplementary Fig. 4c, d).

**Fig. 3:**
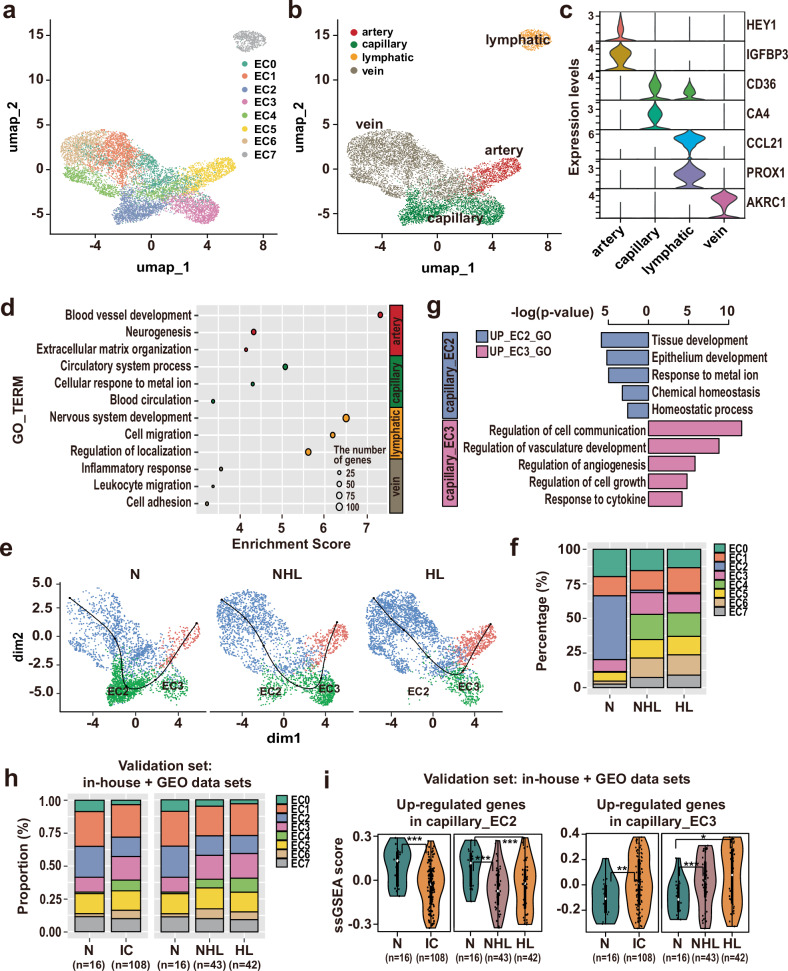
Characterization of endothelial subclusters and their alterations in IC/BPS. **a** Uniform manifold approximation and projection (UMAP) showing eight endothelial cell subclusters. **b** UMAP plot showing the annotation of endothelial cell subclusters. **c** Violin plots showing the canonical marker genes for each endothelial cell subtype. **d** Gene Ontology analysis of each annotated endothelial cell subtype (circles indicate the number of enriched genes). **e** Pseudotime trajectory of endothelial cells across tissue types generated using Slingshot. **f** Stacked bar plots showing the proportions of eight endothelial subclusters aggregated by tissue type. **g** Gene Ontology analysis of the upregulated genes in the capillary E2 and capillary E3 subclusters. **h** Stacked bar plots showing the proportions of the eight endothelial subclusters in the external validation datasets estimated by CIBERSORTx. **i** Violin plots showing single-sample gene set enrichment analysis (ssGSEA) scores of genes upregulated in capillary E2 (left) and E3 (right) in the external validation datasets (*P*-values for box plots were calculated using Student’s *t* test for two groups; **P < 0.05; **P < 0.01; ***P < 0.001). HL, Hunner lesion; IC, interstitial cystitis; N, normal; NHL, non-Hunner lesion.

To further validate these observations, we analyzed two independent public scRNA-seq datasets. In the GSE175526 dataset, the proportion of T_H_17 cells was increased in IC samples compared with control tissues, consistent with our findings. By contrast, the GSE310557 dataset did not show a significant difference in T_H_17 cell proportions between conditions (Supplementary Fig. 4e). However, autoimmune-related gene signatures did not show significant differences between control and IC/BPS samples across these datasets (Supplementary Fig. 4f). To investigate this discrepancy, we examined the characteristics of the control samples used in each dataset. In GSE175526 (ref. ^12^), control samples were derived from non-muscle invasive bladder cancer, whereas in GSE310557 (ref. ^29^), control samples corresponded to pure stress urinary incontinence. Given that these control conditions differ substantially from the normal tissues used in our study, the lack of significant differences in autoimmune-related gene signatures likely reflects underlying differences in baseline tissue characteristics rather than the absence of disease-associated immune activation.

Given the variability observed in public datasets, we sought to further characterize T_H_17 cell biology at the regulatory level by analyzing TF activity using SCENIC analysis^22^. Several TFs, including IRF7, JUNB, PBX4, LEF1, and KLF9, exhibited relatively strong regulon activity in T_H_17 cells and showed increased activity during progression, suggesting potential roles in regulating T_H_17 effector functions (Fig. 2j). To further assess the disease relevance of these TFs, we examined their expression patterns using independent public bulk RNA-seq datasets comparing normal and IC/BPS bladder tissues. *IRF7*, *JUNB*, and *LEF1* expression significantly increased in IC/BPS tissues (Fig. 2k and Supplementary Fig. 4g), whereas *PBX4* and *KLF9* did not show significant differential expression. Lesion-specific comparisons further indicated that *LEF1* expression did not reach statistical significance between N and NHLs (*P* = 0.281) or N and HLs (*P* = 0.058), although HL samples showed a trend toward increased expression (Supplementary Fig. 4g).

Among these TFs, IRF7 and JUNB were prioritized for further analysis because their expression patterns were consistently validated in independent public transcriptomic datasets, whereas other TFs did not show significant or consistent differential expression between normal and IC/BPS tissues. Notably, Tiffany et al. previously demonstrated that JUNB, a component of the activator protein-1 (AP-1) TF, has a pivotal role in regulating T_H_17 cell identity and is essential for the induction and maintenance of T_H_17 effector functions during both acute infection and chronic autoimmunity in mice^30^. To determine whether the inferred TF activities were reflected at the downstream transcriptional level, we performed GSEA using the predicted target gene sets of each regulon. Target genes associated with each regulon showed concordant enrichment patterns with the inferred TF activities, supporting coordinated activation of their downstream transcriptional programs in IC/BPS (Fig. 2l).

### Characterization of endothelial subclusters and their alterations in IC/BPS

To investigate the characteristics of ECs in the IC/BPS microenvironment, we performed unsupervised clustering and identified eight transcriptionally distinct EC subclusters (EC0–EC7) (Fig. 3a). We then annotated these EC subclusters into four major endothelial lineages: arterial, capillary, venous, and lymphatic, based on the expression of known endothelial lineage-specific markers (Fig. 3b, c). For example, *HEY1* and *IGFBP3* were enriched in arterial ECs, *CD36* and *CA4* were enriched in capillary ECs, *CCL21* and *PROX1* were enriched in lymphatic ECs, and *AKRC1* was enriched in venous ECs (Fig. 3c), confirming the distinct identities of each lineage. To independently validate the topographical ordering of these traditional EC subclusters along the artery–capillary–vein axis, we reconstructed a differentiation trajectory using pseudotime analysis (Supplementary Fig. 5a,b). Consistent with a previous study, arterial (*IGFBP3*) and venous (*AKR1C1*) markers were enriched at the opposite ends of the trajectory, whereas capillary markers (*CA4*) were predominantly expressed in the intermediate region, supporting our annotation^31^ (Supplementary Fig. 5c, d).

**Fig. 4:**
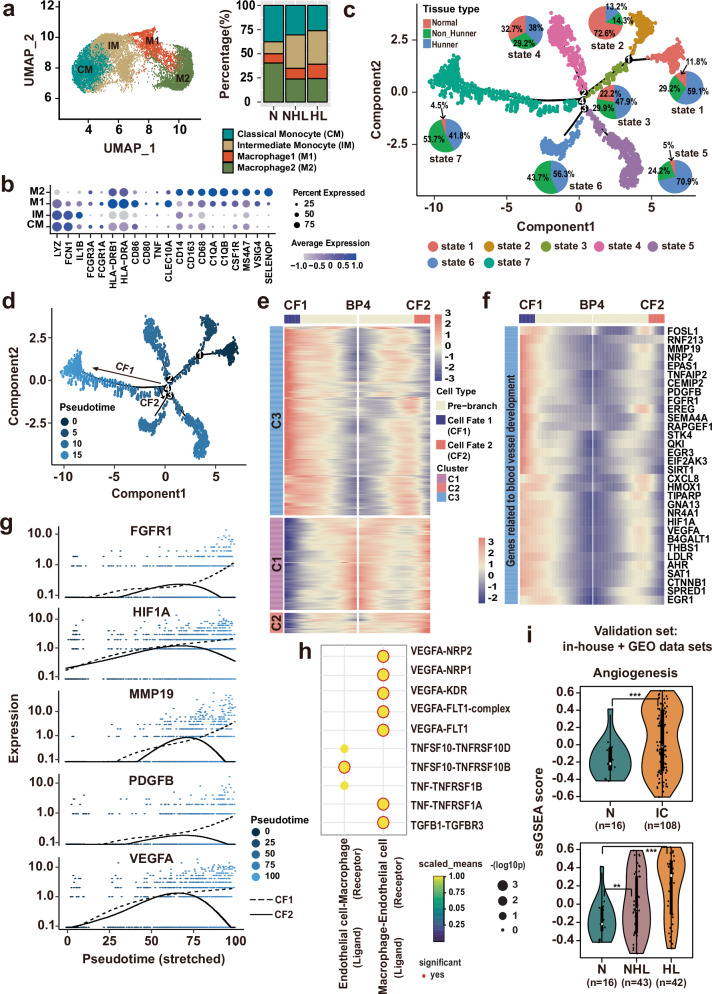
Characterization of monocyte and macrophage subtypes, pseudotime dynamics, and angiogenesis-related interactions. **a** Uniform manifold approximation and projection (UMAP) plot displaying the annotation of monocyte and macrophage subtypes (left) and stacked bar plots showing the proportions aggregated by the tissue type (right). **b** Dot plots showing the expression levels of top marker genes in monocyte and macrophage subtypes (dot size indicates the percentage of cells expressing the gene, and increasing color gradient from gray to purple to blue corresponds to an increasing expression value). Pseudotime trajectory plots of M2 macrophages generated using Monocle2, colored by cell state (part **c**) and pseudotime (part **d**) (pie charts indicate the tissue-type distribution within each state). The state labels represent intermediate transcriptional states along the M2 differentiation trajectory, whereas CF1 and CF2 denote the two terminal cell fates. **e** Heatmap showing gene expression dynamics along pseudotime for clusters C1–C3 at branch point 4 (BP4). **f** Heatmap showing the gene expression dynamics of vascular development-related genes at branch point 4. **g** Expression dynamics of angiogenesis-related genes (*FGFR1*, *HIF1A*, *MMP19*, *PDGFB*, and *VEGFA*) across pseudotime plotted separately for each cell fate branch (dashed line: CF1; solid line: CF2). **h** Analysis of ligand–receptor interactions between macrophages and endothelial cells in IC/BPS samples (color intensity and circle size represent interaction significance based on −log_10_(*P*); interactions labeled as significant indicate *P* < 0.05). **i** Violin plots showing single-sample gene set enrichment analysis (ssGSEA) scores of the angiogenesis gene set in the external validation datasets (*P*-values for violin plots were calculated using Student’s *t* test for two groups: **P < 0.01 and ***P < 0.001). CF1, cell fate 1; CF2, cell fate 2; CM, classical monocyte; HL, Hunner lesion; IC, interstitial cystitis; IM, intermediate monocyte; N, normal; NHL, non-Hunner lesion; VEGF, vascular endothelial growth factor.

Next, we explored the functional characteristics of each EC subtype using GO analysis of the DEGs. Arterial ECs were enriched in pathways involved in vascular development and neurogenesis, whereas venous ECs were enriched in immune-related processes, including inflammatory response, leukocyte migration, and cell adhesion. Lymphatic ECs showed enrichment in pathways related to nervous system development and cell migration. By contrast, capillary ECs were predominantly enriched in core endothelial programs, such as circulatory system processes and blood circulation, while showing limited subtype-specific functional enrichment beyond cellular responses to metal ions. These features were largely shared across endothelial lineages and were indicative of baseline vascular functions (Fig. 3d).

Trajectory analysis revealed notable shifts in the composition and distribution of two capillary subclusters, EC2 and EC3, across tissue types. Particularly, we observed a marked reduction in EC2 cells and an expansion of EC3 cells in IC/BPS tissues than in normal tissues (Fig. 3e, f). To elucidate the functional relevance of these subclusters, we performed GO term enrichment analysis. Capillary EC2 was associated with tissue and epithelial development, response to metal ions, and homeostatic processes, whereas capillary EC3 was enriched in terms of vasculature development, angiogenesis, and cytokine responses (Fig. 3g), indicating distinct biological roles.

Previous studies have reported that urothelial homeostasis is significantly disrupted in the IC/BPS bladder^32^ and that impaired urothelial function is closely linked to disease progression. On the basis of these findings, we hypothesized that the attenuation of homeostatic processes in the capillary EC2 subcluster, along with the enhancement of angiogenic processes, may contribute to IC/BPS pathophysiological progression. To validate these findings, we applied external whole transcriptomic datasets to estimate the proportion of EC types based on the defined EC subclusters. The overall distribution of EC subclusters in these datasets was consistent with our observations (Fig. 3h and Supplementary Fig. 5e–h). Notably, the proportion of EC3 was significantly higher in IC/BPS tissues than in normal tissues, whereas the proportion of EC2 was significantly lower in IC/BPS tissues (Fig. 3h).

To further assess the functional relevance of these capillary EC subtypes, we performed ssGSEA using gene signatures upregulated in EC2 and EC3 cells. The ssGSEA scores for EC2-upregulated genes were significantly higher in normal tissues, whereas the scores for EC3-upregulated genes were higher in IC/BPS tissues (Fig. 3i). Conversely, ssGSEA using downregulated gene sets revealed a significant increase in EC2-associated scores in IC/BPS tissues, whereas no significant change was observed in the EC3-associated scores (Supplementary Fig. 5i). To further validate these findings, we analyzed public scRNA-seq datasets. Although canonical endothelial marker genes such as *PECAM1* and *CDH5* were robustly expressed, lineage-specific markers defining arterial, capillary, venous, and lymphatic endothelial cells showed limited expression in these datasets (Supplementary Fig. 5j), precluding reliable estimation of capillary subtype abundance. Nevertheless, signature-based analysis using EC2-upregulated and EC3-upregulated gene sets revealed that, although the EC2 signature did not differ significantly between conditions, the EC3 signature was significantly enriched in IC/BPS samples, consistent with the angiogenic features observed in our dataset (Supplementary Fig. 5k).

To further distinguish whether these differences reflect shifts in cell composition or transcriptional changes at the per-cell level, we performed tissue-specific differential expression and functional enrichment analyses within each capillary EC subcluster. Within EC2, cells from normal tissues were enriched for gene programs related to homeostatic processes, tissue development, responses to oxidative stress, metabolic processes, and muscle contraction, whereas EC2 cells from IC/BPS tissues showed enrichment of immune response, blood vessel development, and cell–cell communication. By contrast, EC3 exhibited consistent enrichment of angiogenesis-related, vascular development-related, and growth factor response-related pathways across IC/BPS conditions, indicating a stable disease-associated transcriptional program (Supplementary Fig. 5l). Together, these findings suggest that the observed EC2/EC3-associated ssGSEA differences are primarily driven by shifts in relative cell abundance, with only modest transcriptional reprogramming within EC2. Finally, pathway enrichment analysis using hallmark gene sets revealed that transcriptional changes in these ECs were associated with hypoxia, apoptosis, and TNF signaling via NF-κB (Supplementary Fig. 5m). Collectively, these findings suggest that capillary EC subclusters, particularly EC2 and EC3, undergo disease-associated transcriptional reprogramming and functional dysregulation and may actively contribute to the inflammatory and angiogenic remodeling observed in the bladder of patients with IC/BPS.

### Pro-angiogenic macrophage differentiation and vascular remodeling in IC/BPS

To investigate alterations in monocyte and macrophage populations between IC/BPS and normal tissues, we performed re-clustering of cells belonging to the monocyte–macrophage lineage. This analysis revealed four transcriptionally distinct subclusters that were annotated based on the expression of canonical monocyte and macrophage markers. Uniform manifold approximation and projection (UMAP) visualization clearly delineated classical monocytes, intermediate monocytes, and differentiated macrophages, including M1-like and M2-like subtypes (Fig. 4a, b). The proportion of each subset varied depending on the tissue type (Fig. 4a, right).

To investigate the influence of IC/BPS progression on monocyte–macrophage differentiation, we conducted pseudotime trajectory analysis stratified by tissue type. The trajectory structure observed in the NH subtype displayed characteristics of both N and H trajectories, suggesting the presence of an intermediate or transitional differentiation state. Under all tissue conditions, the CM, IM, M1, and M2 cells followed a bifurcating trajectory, culminating in two distinct terminal cell fates (Supplementary Fig. 6a). Notably, the distribution of monocyte–macrophage subclusters and the location of branching points along the trajectories varied across tissue types, indicating that tissue-specific microenvironments may exert unique influences on differentiation pathways. Furthermore, M2 cells consistently bifurcated into two terminal fates in all tissue types, except HLs, implying convergence toward a unified differentiation outcome during disease progression. The trajectories revealed tissue-specific branching points and distinct gene expression patterns (Supplementary Fig. 6b).

To characterize these transcriptional programs, we performed gene expression clustering along a pseudotime trajectory and identified five cell-fate-specific gene modules (C1–C5), each associated with a distinct biological process (Supplementary Fig. 6c). In normal (N) tissues, genes activated in modules C3 and C4 along the cell fate 1 branch were enriched in pathways related to gene expression and regulation of apoptotic processes. By contrast, NH tissues exhibited elevated expression of modules C1, C4, and C5 within the cell fate 1 branch, which are associated with metabolic processes, signal transduction, regulation of programmed cell death, and vascular development. Similarly, in H tissues, modules C3 and C4 of the cell fate 1 branch were enriched for cytokine responses, apoptotic processes, and angiogenesis-related pathways.

Notably, M2 populations in both NHLs and HLs appear to adopt a pro-angiogenic, activated phenotype, potentially contributing to disease pathophysiology. To further investigate this phenomenon, we performed trajectory analysis focussing exclusively on M2 cells across all tissue types. This analysis revealed seven distinct transcriptional states with tissue-specific distributions (Fig. 4c and Supplementary Fig. 6d). Two major terminal branches (CF1 and CF2) emerged from branch point 4 at the distal end of the pseudotime trajectory (Fig. 4d), suggesting divergent differentiation outcomes. To further characterize the transcriptional programs associated with these terminal branches, we performed gene expression clustering along the pseudotime trajectory and identified three gene modules (C1–C3) showing distinct dynamic patterns (Fig. 4e). Functional enrichment analysis revealed that modules C1 and C2, which were predominantly activated along the CF2 branch, were enriched for pathways related to oxidative phosphorylation, immune system processes, and defense responses. By contrast, module C3, associated with the CF1 branch, was enriched for genes involved in regulation of programmed cell death, cytokine response, and blood vessel development (Supplementary Fig. 6e). Heatmaps of branch-dependent gene expression highlighted unique transcriptional programs between the two terminal branches (Fig. 4f). In particular, module C3, associated with the CF1 branch, showed increased expression of angiogenesis-related and vascular development-related genes, including *VEGFA*, *PDGFB*, and *FGFR1* (Fig. 4g).

Consistent with these findings, cell–cell interaction analysis suggested that macrophage–endothelial interactions through the vascular endothelial growth factor (VEGF) and TNF signaling pathways may drive angiogenic remodeling in the IC/BPS microenvironment (Fig. 4h). Moreover, the ssGSEA scores for angiogenesis-related gene sets were significantly higher in the IC/BPS tissues than in the N tissues in the external dataset (Fig. 4i). Given the enrichment of TNF signaling pathways in T_H_17 cells observed in our analysis (Supplementary Fig. 4a), we further investigated whether T_H_17 cells contribute to intercellular communication within the IC/BPS microenvironment. In addition, cell–cell interaction analysis revealed that T_H_17 cells exhibited significantly enhanced TNF-related interactions with endothelial cells and fibroblasts in IC/BPS tissues compared with normal tissues. These findings were further supported by independent public scRNA-seq datasets, which showed a consistent pattern of increased TNF-mediated interactions (Supplementary Fig. 7a,b). Taken together, these findings suggest that distinct M2 cell trajectories, together with T_H_17-mediated inflammatory signaling, engage in angiogenic programs and may cooperate with ECs to drive vascular remodeling in the IC/BPS microenvironment.

### SMC phenotypic shifts and EDN1-mediated signaling underlie increased contractility in IC/BPS

**Fig. 5:**
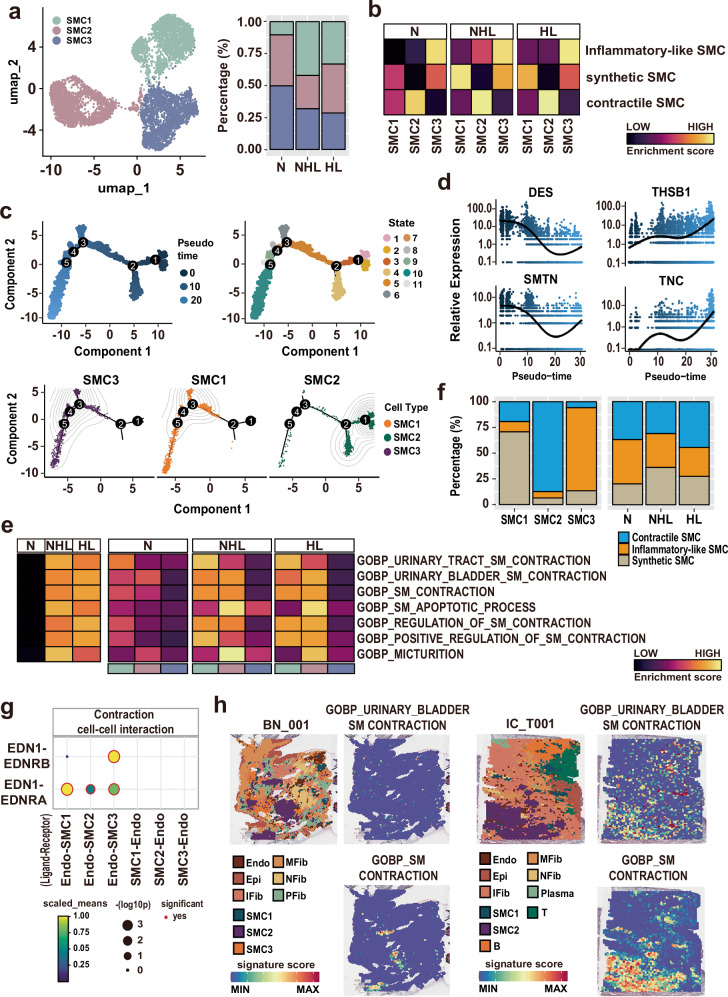
SMC phenotypic shifts and EDN1-mediated signaling underlie increased contractility in IC/BPS. **a** Uniform manifold approximation and projection (UMAP) plot displaying the annotation of smooth muscle cell (SMC) subtypes (left) and stacked bar plots showing their proportions aggregated by the tissue type (right). **b** Heatmap of gene set enrichment analysis scores of SMC phenotypes, including inflammatory-like, synthetic, and contractile SMCs. **c** Pseudotime trajectory plots of SMCs generated using Monocle2, colored by pseudotime (top left) and cell state (top right); the bottom panel shows pseudotime trajectories stratified by tissue type. **d** Loess-smoothed expression patterns of SMC phenotype marker genes along the pseudotime trajectory. **e** Heatmap of gene set enrichment analysis scores of contraction-related gene sets across tissue types and SMC subtypes. **f** Stacked bar plots showing the proportions of SMC phenotypes inferred using the nearest template prediction (NTP) algorithm. **g** Ligand–receptor interaction analysis between endothelial cells and SMCs in IC/BPS tissues (color intensity and circle size represent interaction significance based on −log_10_(*P*); interactions labeled as significant indicate *P* < 0.05). **h** Spatial transcriptomic map of control (left) and IC/BPS (right) tissues, colored by cell type based on conditional autoregression-based deconvolution analysis. Spatial plots showing contraction-related signature scores were calculated using the AddModule Score. HL, Hunner lesion; IC/BPS, interstitial cystitis/bladder pain syndrome; N, normal; NHL, non-Hunner lesion.

To characterize SMC heterogeneity in the bladder across IC/BPS tissue types, we performed unsupervised clustering of SMCs and identified three transcriptionally distinct SMC subclusters (SMC1–3) (Fig. 5a and Supplementary Fig. 8a,b). The relative abundance of these clusters varied markedly with the disease condition. SMC2 and SMC3 were predominant in normal (N) tissues, whereas SMC1 was enriched in IC/BPS tissues, suggesting disease-associated remodeling of the SMC compartment (Fig. 5a and Supplementary Fig. 8c).

Given that SMCs are known to undergo phenotypic transitions in pathological conditions such as atherosclerosis^20,21^, we performed ssGSEA to further characterize each SMC subcluster. This analysis revealed that SMC2 displayed features of contractile SMCs, whereas SMC1 and SMC3 exhibited transcriptional signatures associated with synthetic and inflammation-like phenotypes, respectively (Fig. 5b). Pseudotime trajectory analysis demonstrated a continuous transition from SMC2 (contractile) to SMC1 (synthetic) to SMC3 (inflammatory-like), indicating dynamic phenotypic plasticity among SMCs in IC/BPS (Fig. 5c). This transition was supported by gradual changes in the expression of phenotype-specific markers, such as *DES*, *THBS1*, *SMTN*, and *TNC*^20,21^ (Fig. 5d).

To assess the functional activation of SMC-related biological processes under disease conditions, we performed ssGSEA using gene sets associated with smooth muscle contraction. The enrichment of contraction-related gene sets, including urinary tract smooth muscle contraction, urinary bladder smooth muscle contraction, and positive regulation of smooth muscle contraction, was significantly higher in IC/BPS tissues than in normal tissues (Fig. 5e and Supplementary Fig. 8d). Despite the well-established association of synthetic SMCs with reduced contractility and increased proliferative/migratory potential, SMC1, which predominantly displays a synthetic phenotype, was notably enriched in contraction-related pathways. This suggests that a subset of synthetic SMCs may retain their functional contractile capacity. We applied the nearest template prediction algorithm to further delineate the phenotypic identity of each SMC subcluster. This classification revealed that SMC2 consisted mainly of contractile SMCs (88%), SMC3 was enriched in inflammatory-like SMCs (81%), and SMC1 primarily comprised synthetic SMCs (71%) with a minor population of contractile SMCs (19%) (Fig. 5f). These findings support the idea that SMC1 includes a functionally hybrid population that retains a partial contractile potential.

Validation using external whole transcriptome datasets provided partial evidence for the existence of distinct SMC phenotypes and their associated functional pathways (Supplementary Fig. 8e,f). Although contraction-related gene sets did not show statistically significant overall enrichment, the positive regulation of the smooth muscle contraction gene set was activated in the IC/BPS samples (Supplementary Fig. 8e). Moreover, the expression of genes associated with the synthetic SMC phenotype was elevated in the IC/BPS samples (Supplementary Fig. 8f), consistent with the expansion of synthetic SMCs under pathological conditions.

We further explored the intercellular communication involving SMCs through ligand–receptor analysis, which revealed enhanced endothelial–SMC signaling in IC/BPS tissues. In particular, the EDN1–EDNRA/EDNRB axis — a well-known mediator of smooth muscle contraction^33^ — was prominently upregulated (Fig. 5g). External whole transcriptome data confirmed that *EDN1* expression was significantly increased in IC/BPS samples, whereas *EDNRA* and *EDNRB* expression remained unchanged (Supplementary Fig. 8g), highlighting *EDN1* as a potential molecular driver of hypercontractility.

Owing to limitations in spatial transcriptomic data quality and cell-type diversity, such as BN_002 consisting of 99% SMC2 and IC_T002 exhibiting a low number of detected features, only the BN_001, IC_T001, and IC_T003 samples were included in the spatial analyses (Supplementary Fig. 9a–c). Spatial mapping revealed increased expression of contraction-related gene sets in specific regions of IC_T001 and IC_T003, along with the localization of distinct SMC subtypes, particularly SMC2, compared with BN_001 (Fig. 5h and Supplementary Fig. 9d–f). Notably, spatial cell–cell interaction analysis further supported differential EDN1 signaling: BN_001 exhibited EDN1–EDNRB interactions, whereas IC_T001 showed both EDN1–EDNRA and EDN1–EDNRB interactions (Supplementary Fig. 9g,h). Owing to limitations in the spatial features of IC_T003, cell–cell interactions could not be detected. These findings suggest that EDN1–EDNRA/ EDNRB signaling between ECs and SMCs is a potential driver of increased contractility in ICs/BPSs.

To further validate these findings at the single-cell level, we analyzed independent public scRNA-seq datasets. In contrast to the endothelial analysis, SMC subtypes were well resolved and showed a distribution pattern consistent with our dataset, supporting the robustness of SMC classification (Supplementary Fig. 10a,b). In addition, contraction-related gene sets were consistently enriched in IC/BPS samples, with particularly high activation observed in the SMC2 subtype (Supplementary Fig. 10c). Furthermore, cell–cell interaction analysis using public scRNA-seq dataset recapitulated key signaling patterns identified in our dataset, including endothelial–SMC communication axes representing robust and conserved features of IC/BPS pathophysiology (Supplementary Fig. 10d,e). To extend these observations into a spatial context and achieve higher resolution validation, we generated Visium HD data from six samples, including three normal and three IC/BPS tissues. One normal sample (BN_004) was excluded from downstream analysis owing to the absence of smooth muscle cells, with a predominant B cell population (Supplementary Fig. 10f). Using the remaining five samples, high-resolution cell–cell interaction analysis revealed patterns consistent with the endothelial–SMC signaling interactions identified in both scRNA-seq and standard Visium analyses (Supplementary Fig. 10g). Although cell–cell interactions were detectable in one normal sample (BN_003), they did not reach statistical significance. By contrast, IC/BPS samples showed more consistent interaction patterns, with two samples (IC_T009 and IC_T010) exhibiting significant interactions, whereas one sample (IC_T011) did not reach statistical significance. In addition, spatial mapping confirmed that contraction-related gene sets were more strongly activated in IC/BPS tissues (Supplementary Fig. 10h), further supporting the robustness and spatial consistency of SMC-associated contractile activation in IC/BPS.

### Chronic pain associated with fibroblasts and SMCs in IC/BPS

**Fig. 6:**
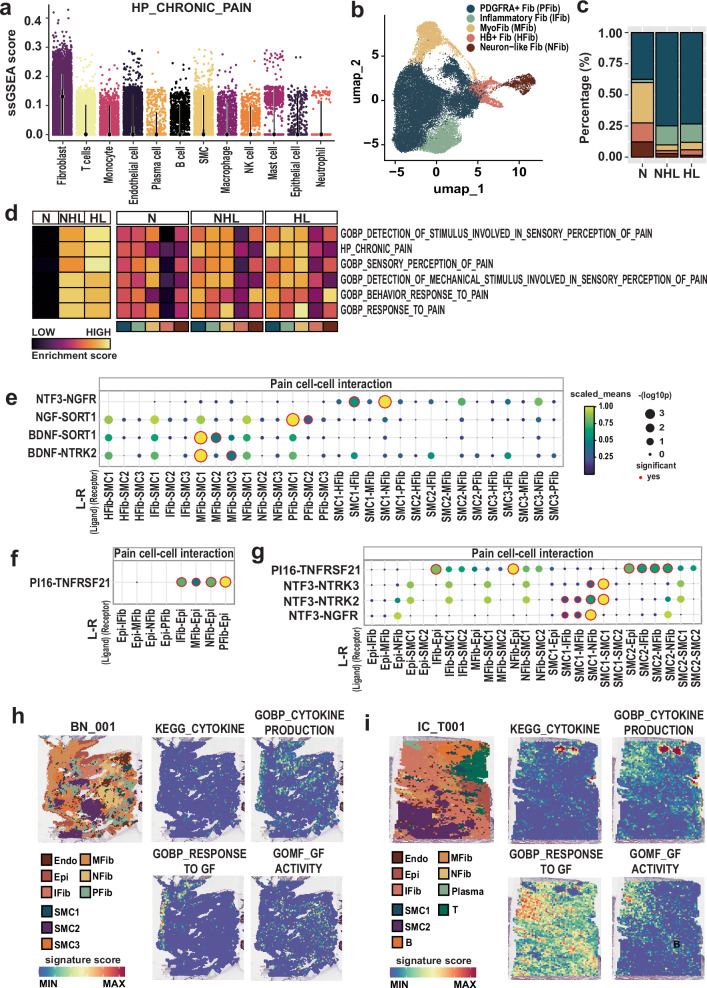
Chronic pain associated with fibroblasts and SMC in IC/BPS. **a** Box plots showing sample gene set enrichment analysis (ssGSEA) scores of the HP_CHRONIC_PAIN gene set across the 12 major cell types. **b**, **c** Uniform manifold approximation and projection (UMAP) plot illustrating the annotation of fibroblast subtypes, including PDGFRA+ fibroblasts (PFib), inflammatory fibroblasts (IFib), myofibroblasts (MFib), hemoglobin-expressing fibroblasts (HFib), and neuron-like fibroblasts (NFib). **b** Stacked bar plots showing the proportion of each fibroblast subtype across tissue types (part **c**). **d** Heatmap of GSEA enrichment scores of pain-related gene sets across the tissue and fibroblast subtypes. **e** Ligand–receptor interaction analysis between fibroblasts and smooth muscle cells (SMCs) inferred from single-cell RNA-seq data in IC/BPS tissues. Ligand–receptor pairs identified from spatial transcriptomic data in control (BN_001) (part **f**) and IC/BPS (IC_T001) (part **g**) tissues (circle size and color intensity represent statistical significance of interactions (−log₁₀ *P*); interactions marked as significant indicate *P* < 0.05). Spatial transcriptomic maps of control (part **h**) and IC/BPS (part **i**) tissues, colored by the cell type predicted using conditional autoregression-based deconvolution. Spatial plots displaying cytokine-related and growth factor-related signature scores were generated using AddModuleScore. HL, Hunner lesion; IC/BPS, interstitial cystitis/bladder pain syndrome; KEGG, Kyoto Encyclopedia of Genes and Genomes; N, normal; NHL, non-Hunner lesion; NK, natural killer.

Chronic pain is a well-recognized pathological feature of IC/BPS. To explore the cell-type-specific contributions to this phenotype, we performed ssGSEA across all cell types. Fibroblasts exhibited the highest enrichment of the HP_CHRONIC_PAIN signature (Fig. 6a), indicating that they are the key contributors to chronic pain in IC/BPS^34^.

To further investigate the fibroblast-specific alterations between IC/BPS and normal tissues, we re-clustered the fibroblast populations (Supplementary Fig. 11a). This analysis identified five transcriptionally distinct subclusters annotated based on canonical marker gene expression^12,13,35^ (Supplementary Fig. 11b). Notably, a subset of neuron-like fibroblasts expressed high levels of neuronal genes such as *GPM6B* and *NRXN1* (ref. ^35^), whereas inflammatory fibroblasts were characterized by elevated expression of *IL6*, *CXCL14*, and other pro-inflammatory mediators, suggesting functional specialization within the fibroblast compartment.

UMAP visualization revealed five fibroblast subtypes: PDGFRA+ fibroblasts (PFib), inflammatory fibroblasts (IFib), myofibroblasts (MFib), hemoglobin-expressing fibroblasts (HFib), and neuron-like fibroblasts (NFib) (Fig. 6b). The relative abundances of these subtypes varied between the IC/BPS and normal tissues. Particularly, IFib was markedly increased in IC/BPS samples, whereas MFib was more prevalent in normal tissues (Fig. 6c). To characterize the functional roles of the expanded PFib and IFib populations in IC/BPS, we performed GO enrichment analysis. Both subsets were enriched in immune-related processes; notably, IFib showed significant enrichment in lymphocyte chemotaxis, whereas PFib was more associated with adaptive immune responses (Supplementary Fig. 11c,d). To further define their immune-related features in the IC/BPS context, we performed ssGSEA using cytokine-related gene signatures. These signatures were elevated in IC/BPS tissues, with both IFib and PFib exhibiting higher enrichment scores compared with other fibroblast subtypes (Supplementary Fig. 11e). These findings support the emerging concept that fibroblasts act as active immune effectors, displaying functional heterogeneity in chronic inflammatory conditions^36^. Furthermore, given that inflammatory mediator secretion by pathological fibroblasts has been linked to pain severity in chronic disease models^37^, our results suggest that the expansion of IFib and PFib may contribute to the inflammatory microenvironment of IC/BPS. Collectively, these data indicate that these fibroblast subsets may have a key role in driving inflammatory and pain-associated processes in IC/BPS.

To deepen our understanding of fibroblast-mediated pain signaling, we evaluated six pain-related gene sets in the MSigDB database. All six gene sets were significantly enriched in IC/BPS tissues compared with normal tissues (Fig. 6d and Supplementary Fig. 11f). Although validation using external whole transcriptomic datasets did not yield statistically significant ssGSEA scores, the overall expression trends indicated the activation of pain-related gene sets in the IC/BPS samples (Supplementary Fig. 11g). Collectively, these findings support the central role of fibroblasts in mediating chronic pain in patients with IC/BPS.

To identify the specific signaling axes mediating fibroblast-driven pain responses, we analyzed cell–cell communication. The well-known PI16–TNFRSF21 pain signaling pathway was predominantly detected between fibroblasts and epithelial cells in both normal and IC/BPS tissues^38,39^ (Supplementary Fig. 12a). Ligand–receptor interaction analysis revealed several neurotrophin-related signaling pairs enriched in IC/BPS fibroblasts and SMCs, including NTF3–NTRK2/NTRK3 and NGF–SORT1 (refs. ^40,41^) (Supplementary Fig. 12b).

Further analysis of intercellular interactions between fibroblasts and SMC subclusters revealed key neurotrophic factor axes, such as NTF3–NGFR, NGF/BDNF–SORT1, and BDNF–NTRK2 (refs. ^42,43^), suggesting that MFib and SMC1 may engage in paracrine signaling, contributing to nociceptive pathways (Fig. 6e). Moreover, spatial cell–cell interaction analysis confirmed the presence of PI16–TNFRSF21 interactions in both BN_001 and IC_T001 tissues (Fig. 6f, g). NTF3–NTRK2/NTRK3 and NTF3–NGFR interactions were detected in IC_T001 tissues and were particularly enriched in the SMC1 and SMC2 subpopulations.

Although SMCs are not typically involved in nociception owing to their lack of neuronal features, previous studies have suggested that various cell types can acquire altered phenotypes under the influence of steroid hormones, cytokines, and growth factors^44–47^. To test this hypothesis, we utilized spatial transcriptome analysis to assess cytokine-related and growth factor-related gene sets from MSigDB. Spatial data revealed that these gene sets were significantly enriched in T cells and IFib around SMC1 and SMC2 in IC_T001 and IC_T003 tissues compared with BN_001 tissues (Fig. 6h, i and Supplementary Fig. 12c). These findings suggest that prolonged exposure to growth factors and inflammatory cues facilitates the partial transdifferentiation of SMCs into neuron-like phenotypes, potentially contributing to the pain phenotype observed in IC/BPS.

To further validate these findings at the single-cell level, we analyzed public scRNA-seq datasets. Although the six pain-related gene sets exhibited heterogeneous enrichment patterns, with approximately half enriched in normal tissues and the other half enriched in IC/BPS tissues, this variability likely reflects differences in normal cohort composition across datasets (Supplementary Fig. 13a). Importantly, cell–cell interaction analyses consistently recapitulated our primary findings, indicating robust activation of pain-associated intercellular signaling networks in IC/BPS (Supplementary Fig. 13b–d). To extend these findings with higher spatial resolution, we analyzed Visium HD data. In normal tissues, cell–cell interactions were detected in two samples (BN_003 and BN_004) but did not reach statistical significance, whereas no detectable interactions were observed in BN_005 (Supplementary Fig. 13e). By contrast, all three IC/BPS samples (IC_T009, IC_T010, and IC_T011) exhibited significant pain-associated cell–cell interactions (Supplementary Fig. 13f,g). Notably, although conventional Visium analysis primarily identified PI16–TNFRSF21 interactions between epithelial cells and fibroblasts, Visium HD analysis revealed a broader spectrum of interactions across multiple cell subtypes, particularly in IC/BPS tissues (Supplementary Fig. 13f,g). Moreover, spatial mapping of cytokine-related and growth factor-related gene sets revealed markedly higher activation in IC/BPS tissues compared with normal tissues, supporting enhanced inflammatory and paracrine signaling within the IC/BPS microenvironment (Supplementary Fig. 14a, b). Consistent with these findings, TNF-related signaling interactions involving T_H_17 cells and endothelial cells were significantly enriched in IC/BPS tissues, and spatial analyses from both Visium and Visium HD datasets confirmed increased activation of TNF signaling pathways (Supplementary Fig. 15a,b). These results suggest that T_H_17-driven TNF-mediated inflammation may contribute to both enhanced contractile responses and pain-associated signaling through interactions with stromal and vascular cell types. Collectively, our integrative single-cell and spatial analyses reveal a coordinated multicellular network in which fibroblast–SMC communication, neurotrophic signaling, and T_H_17-associated TNF-driven inflammatory interactions converge to shape the pathological microenvironment underlying IC/BPS.

## Discussion

To our knowledge, this is the first single-cell and spatial transcriptomic study to dissect and compare the cellular landscapes of HLs and NHLs in individuals diagnosed with IC/BPS. By leveraging high-resolution transcriptomic profiling of full-thickness bladder tissues, our study provides an unprecedented view of the spatial and cellular characteristics underlying IC/BPS pathogenesis. This integrated understanding of cellular states and their interactions offer therapeutic opportunities for modulating immune responses, vascular remodeling, and pain signaling in patients with IC/BPS.

Our findings highlight a central role for T_H_17 cells in the pathogenesis of IC/BPS, particularly within HLs. Beyond their increased abundance, T_H_17 cells in HLs exhibit a distinct transcriptomic profile characterized by the activation of inflammatory, cytokine, and TNF signaling pathways, along with the enrichment of autoimmune-associated gene signatures. These features collectively support the involvement of autoimmune mechanisms in disease etiology.

Mechanistically, T_H_17 cells are known to mediate pathogenic effects through the secretion of IL-17 and TNF, which promote endothelial activation, pathological angiogenesis, and chronic tissue remodeling^48,49^. The enrichment of these pathways in HL-derived T_H_17 cells suggests that they may represent a source of inflammatory milieu that triggers the downstream EC and SMC transitions we observed. In addition, the upregulation of TFs such as IRF7 and JUNB provides molecular support for a sustained T_H_17 effector program. Notably, JUNB has been implicated in the maintenance of T_H_17 functionality in chronic autoimmune conditions^30,50^, reinforcing the idea that these cells actively contribute to persistent inflammation rather than serving as transient infiltrates.

Importantly, our study also suggests a potential link between T_H_17 activation and chronic pain, a hallmark symptom of IC/BPS. Previous studies indicate that T_H_17-derived cytokines, particularly IL-17, can contribute to nociceptive sensitization through neuroimmune interactions^51,52^. In line with this, the enrichment of TNF and inflammatory signaling pathways in T_H_17 clusters, together with their potential interactions to fibroblasts and SMCs, raises the possibility that T_H_17 cells may lower the activation threshold of bladder nociceptors. Although direct cytokine measurements and functional perturbations were not performed, the transcriptomic evidence presented here supports a model in which T_H_17-driven immune responses contribute to both vascular and smooth muscle remodeling and sensory hypersensitivity in IC/BPS.

EC profiling revealed notable heterogeneity and disease-associated shifts in endothelial subclusters. We observed a reduction in EC2 cells, which are implicated in epithelial and tissue homeostasis, along with an expansion of EC3 cells enriched for angiogenic and inflammatory pathways, indicating a phenotypic transition of ECs from a homeostatic to pro-inflammatory and pro-angiogenic states in IC/BPS. This endothelial transition appears to be driven, at least in part, by inflammatory signaling and hypoxic conditions. Particularly, the activation of hypoxia, apoptosis, and TNF–NF-κB signaling pathways in ECs reinforces the presence of a hypoxia-driven angiogenic niche. Hypoxia activates the NF-κB pathway, which in turn upregulates pro-angiogenic and inflammatory genes, thereby sustaining vascular remodeling and immune activation^53–55^. These findings support the notion that inflammation and hypoxia cooperate to reprogram EC states and amplify the pathological cycle of angiogenesis in IC/BPS.

Our analysis of the monocyte–macrophage lineage revealed a complex differentiation landscape shaped by the local microenvironment. Particularly M2 cells, in IC/BPS, adopt a distinct pro-angiogenic transcriptional program marked by elevated expression of *VEGFA*, *PDGFB*, and *FGFR1*. These changes imply that M2 cells are not merely immunoregulatory but also key contributors to pathological vascular remodeling, likely through enhanced VEGF-mediated and TNF-mediated interactions with ECs^56^. This pro-angiogenic phenotype suggests a potential mechanism by which aberrant angiogenesis is sustained in the IC/BPS microenvironment^53^.

Simultaneously, we observed marked phenotypic plasticity in the bladder SMCs. IC/BPS tissues showed a substantial increase in synthetic SMCs and a concurrent decrease in contractile SMCs. The predominance of synthetic SMCs, which represent a dedifferentiated and plastic state, suggests that SMCs undergo phenotypic switching in response to local environmental cues. Such cues may include a combination of growth factors, immune-inflammatory molecules, and metabolic factors^20,21^ — all of which are hallmarks of the IC/BPS tissue milieu. In addition, single-cell and spatial transcriptomic analyses revealed the upregulation of the EDN1–EDNRA/EDNRB signaling axis between ECs and SMCs, implicating EDN1 as a key molecular driver of increased smooth muscle tone and impaired bladder function in IC/BPS^33^. This endothelial-to-SMC signaling pathway may contribute to increased muscle contractility and bladder hypersensitivity, further exacerbating urinary symptoms and pain. These findings suggest that targeting the EDN1 signaling cascade is a promising therapeutic strategy for alleviating smooth muscle hyperactivity and restoring bladder homeostasis in patients with IC/BPS.

Chronic pain, a hallmark symptom of IC/BPS, is closely associated with the activity of specific fibroblasts in the bladder microenvironment. In our analysis, fibroblasts showed the highest enrichment of the chronic pain gene signature, suggesting their central role in nociceptive modulation (Fig. 6a). These fibroblasts express key components of the neurotrophin signaling pathway and engage in ligand–receptor interactions, such as the PI16–TNFRSF21 and NTF3–NTRK2/NTRK3 axes, which are known to influence neuronal signaling and pain perception^38–42^. This indicates that fibroblasts may act as critical mediators of neuroimmune crosstalk and contribute directly to the generation and maintenance of chronic pain in patients with IC/BPS.

Interestingly, we also observed that synthetic-like SMC1 cells participated in neurotrophic signaling and spatially colocalized with IFib and cytokine-producing T cells. This anatomical and functional proximity suggests that prolonged inflammatory stimuli promote the partial transdifferentiation of SMCs into neuron-like or neuromodulatory phenotypes. Such phenotypic plasticity may further amplify nociceptive signaling pathways within the bladder wall. Our data support a model in which immune–fibroblast–SMC interactions drive maladaptive tissue remodeling and sensory hypersensitivity, ultimately contributing to the chronic pain condition observed in patients with IC/BPS.

Taken together, our findings elucidate a complex multicellular network underpinning IC/BPS pathogenesis, characterized by T_H_17-driven inflammation and autoimmunity, endothelial dysfunction, pathological angiogenesis mediated by pro-angiogenic macrophages, SMC phenotypic remodeling linked to bladder hypercontractility, and a fibroblast–SMC axis that contributes to chronic pain. These insights offer new potential therapeutic targets, including the modulation of T_H_17 activity, EDN1 signaling, macrophage-driven angiogenesis, and fibroblast-mediated nociceptive pathways, which could collectively improve the management of inflammation, bladder dysfunction, and pain in patients with IC/BPS.

Despite the comprehensive insights provided by our single-cell and spatial transcriptomic analyses, this study has several limitations. First, the relatively small sample size, particularly the limited number of healthy control tissues, may limit the generalizability of our findings. This reflects the clinical and ethical challenges of obtaining full-thickness bladder tissue, especially from well-characterized IC/BPS lesions. Our use of partial cystectomy specimens enabled precise paired sampling of HL and NHL, providing high anatomical resolution while inherently limiting sample size. To mitigate this, we validated our findings using independent external datasets and high-resolution spatial transcriptomics, supporting the robustness of our results. Second, although we successfully characterized a broad spectrum of cell populations, neuronal cells were not detected, limiting our ability to directly investigate pain-related mechanisms at the neuronal level. Third, spatial transcriptomic analyses of discovery cohort were conducted on a limited number of samples (*n* = 3) owing to data quality constraints and restricted cell-type diversity. To address this limitation, we generated additional Visium HD data as an independent validation cohort, enabling higher-resolution spatial characterization and validation of key findings. Nevertheless, current spatial transcriptomic approaches do not yet achieve true single-cell resolution, underscoring the need for next-generation platforms such as Xenium for more precise spatial validation. Fourth, all IC/BPS samples were obtained from patients who had undergone supratrigonal cystectomy with augmentation cystoplasty, a procedure typically performed in severe cases. This may have resulted in sample bias and limited the generalizability of the findings to a broader clinical spectrum of IC/BPS. However, the inclusion of histologically normal-appearing NHLs and the use of full-thickness bladder tissues, unlike superficial cold cup biopsies commonly used in other studies, enhanced the biological relevance and depth of our analysis. Finally, functional validation of key molecular targets, such as EDN1, JUNB, and components of neurotrophin signaling, through in vitro and in vivo experiments is necessary to confirm their mechanistic roles and therapeutic relevance in IC/BPS.

## Supplementary information

Supplementary Information

## Data Availability

The data used in this study are available from the corresponding author upon request (dr.minyong.kang@gmail.com). The experimental protocols and data analysis pipeline used in our study followed the official 10× Genomics and Seurat websites. No custom code was used for data analysis in this study.
