# Supplementary material for: Integrated single-cell and spatial transcriptomics reveal stromal–immune–vascular crosstalk in patients with interstitial cystitis/bladder pain syndrome
Source: Exp Mol Med. 2026 Jul 3;58(7):2235–51. doi: 10.1038/s12276-026-01768-2 (PMC13434039; doi:10.1038/s12276-026-01768-2)
Supplement: Supplementary file 1 [file 12276_2026_1768_MOESM1_ESM.pdf]

Supplementary Figures and Tables

Supplementary Fig. 1

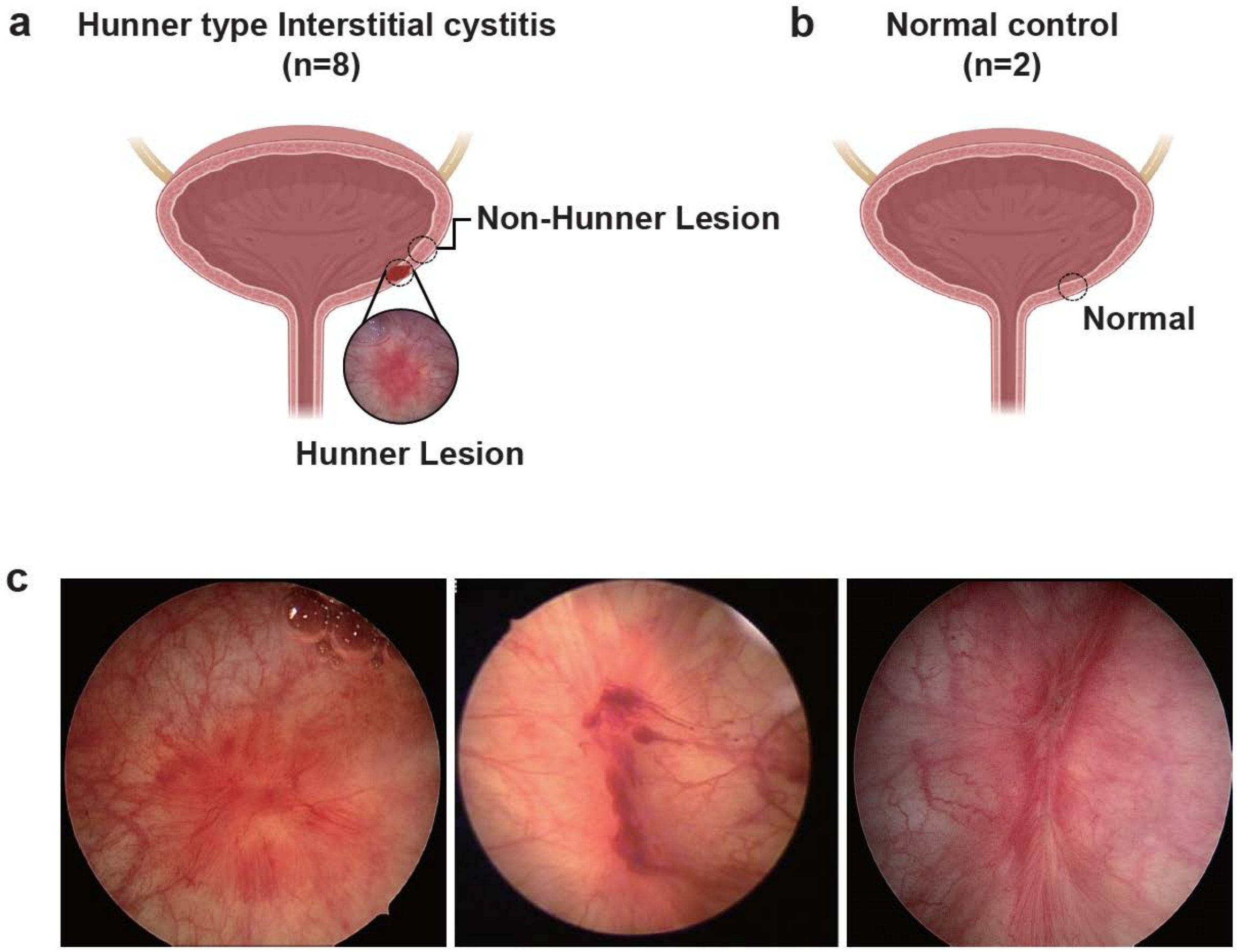

Supplementary Fig. 1. Representative bladder regions and cystoscopic findings in patients with Hunner-type IC/BPS and controls. a. Schematic representation of bladder sampling sites in patients with Hunner-type interstitial cystitis/bladder pain syndrome (IC/BPS), including both Hunner and non-Hunner lesion areas. **b.** Normal bladder tissue was obtained from control participants without IC/BPS. **c.** Representative cystoscopic images of Hunner lesions showing mucosal erythema, neovascularization, and ulceration, typical of Hunner-type IC/BPS.

Supplementary Fig. 2

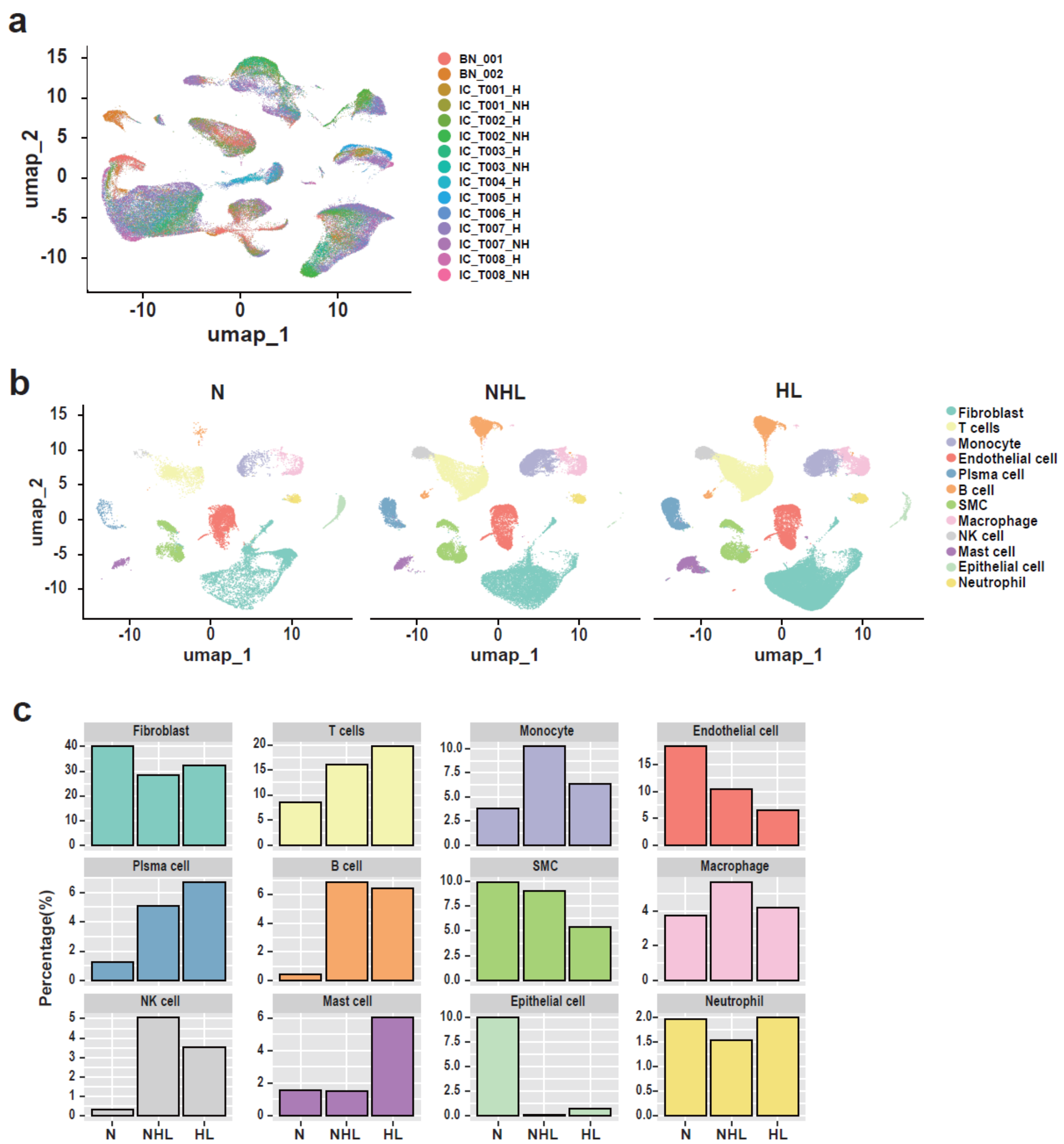

Supplementary Fig. 2. Overall distribution of single-cell transcriptomic profiles in IC/BPS.

a. UMAP plot before batch correction **b.** UMAP plots showing the annotation of the 12 major cell types separated by tissue type. **c.** Bar plots displaying the percentages of 12 major cell types across tissue types. (UMAP, uniform manifold approximation and projection; SMC, smooth muscle cell; NK, natural killer; N, normal; NHL, Non-Hunner lesion; HL, Hunner lesion)

Supplementary Fig. 3

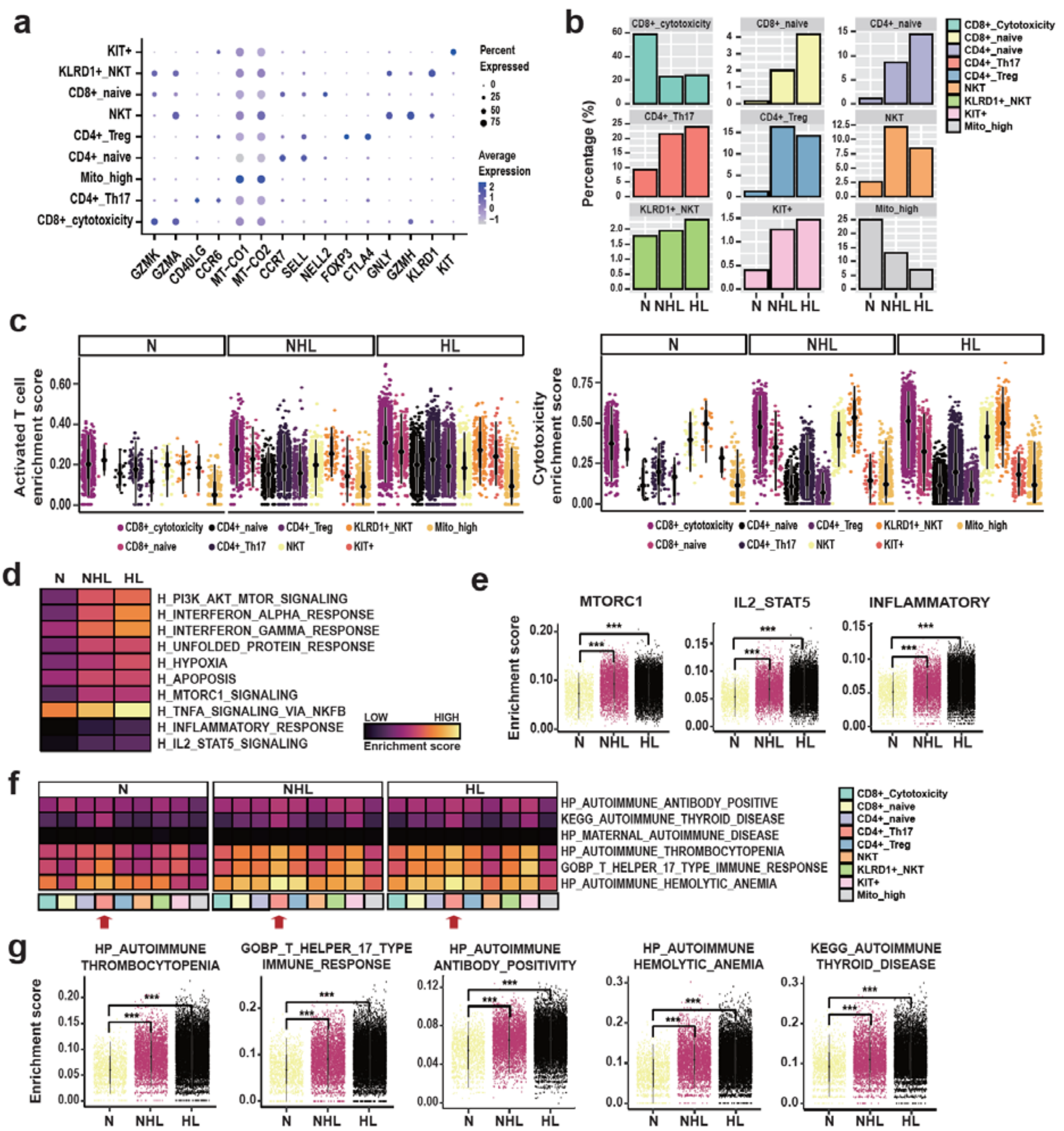

Supplementary Fig. 3. Functional characterization of T cell sub-clusters. a. Average expression dot plot shows the top marker genes in the T cell sub-clusters (the size of the circles indicates the percentage of cells expressing the gene, and the increasing color gradient from gray to purple to blue corresponds to increasing expression values). **b.** Bar plots showing the

percentages of T cell sub-clusters across tissue types. **c.** Box plots illustrating ssGSEA enrichment scores of activated T cell- and cytotoxicity- related gene sets across cell types. **d.** Heatmap of GSEA scores of Hallmark gene sets. **e.** Box plots illustrating ssGSEA enrichment scores of MTORC1, IL2_STAT5, and Inflammatory response (INFLAMMATORY) signaling pathways across tissue types. **f.** Heatmap of GSEA scores for autoimmune-related gene sets across T cell subclusters and tissue types (Red arrows indicate Th17 cells). **g.** Box plots illustrating ssGSEA enrichment scores of autoimmune-related gene sets across tissue types. (N, normal; NHL, Non-Hunner lesion; HL, Hunner lesion, *p*-values for box plots were calculated using Student's *t-test* for two groups; *** $p < 1 \times 10^{-4}$)

Supplementary Fig. 4

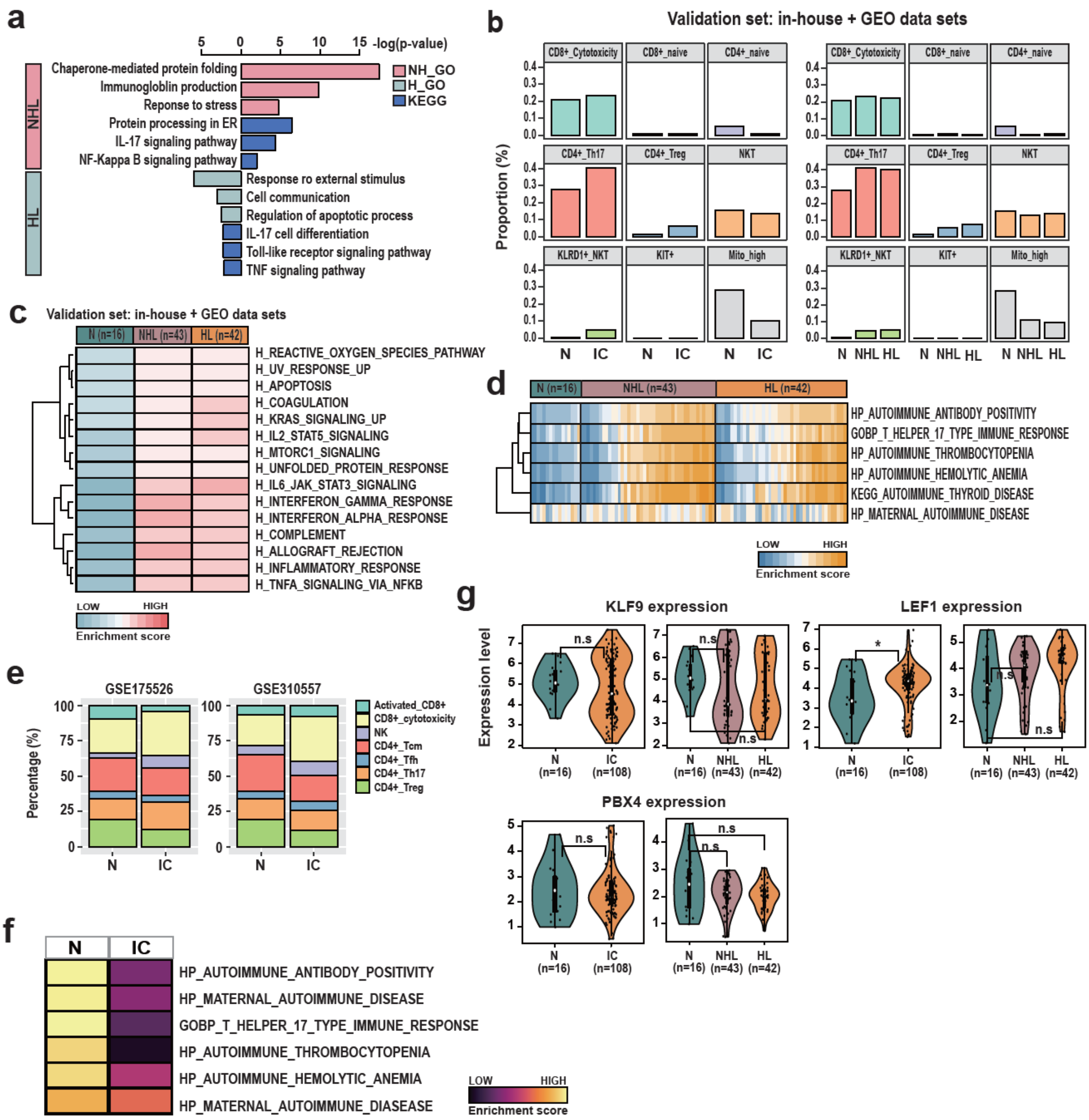

Supplementary Fig. 4. Validation using external datasets. a. GO and KEGG pathway analyses using differentially expressed genes in Th17 cells between NHL and HL tissues. **b.** Bar plots showing the percentage of T cell sub-clusters across tissue types using external validation dataset. **c.** Heatmap of ssGSEA scores of Hallmark gene sets using external validation datasets. **d.** Heatmap of GSEA scores for autoimmune-related gene sets using external validation datasets. **e.** Stacked bar plots showing the proportion of seven T cell sub-

clusters using public scRNA-seq datasets. **f.** Heatmap of ssGSEA scores of autoimmune related gene sets using public scRNA-seq datasets. **g.** Violin plots showing the expression levels of *KLF9*, *LEF1* and *PBX4* in the external validation datasets. (N, normal; NHL, Non-Hunner lesion; HL, Hunner lesion; IC, Interstitial Cystitis, *p*-values for box plots were calculated using Student's *t-test* for two groups; Significance was determined using Student′s *t*-test. NS, not significant; *$p < 0.05$; **$p < 0.01$; ***$p < 0.001$)

Supplementary Fig. 5

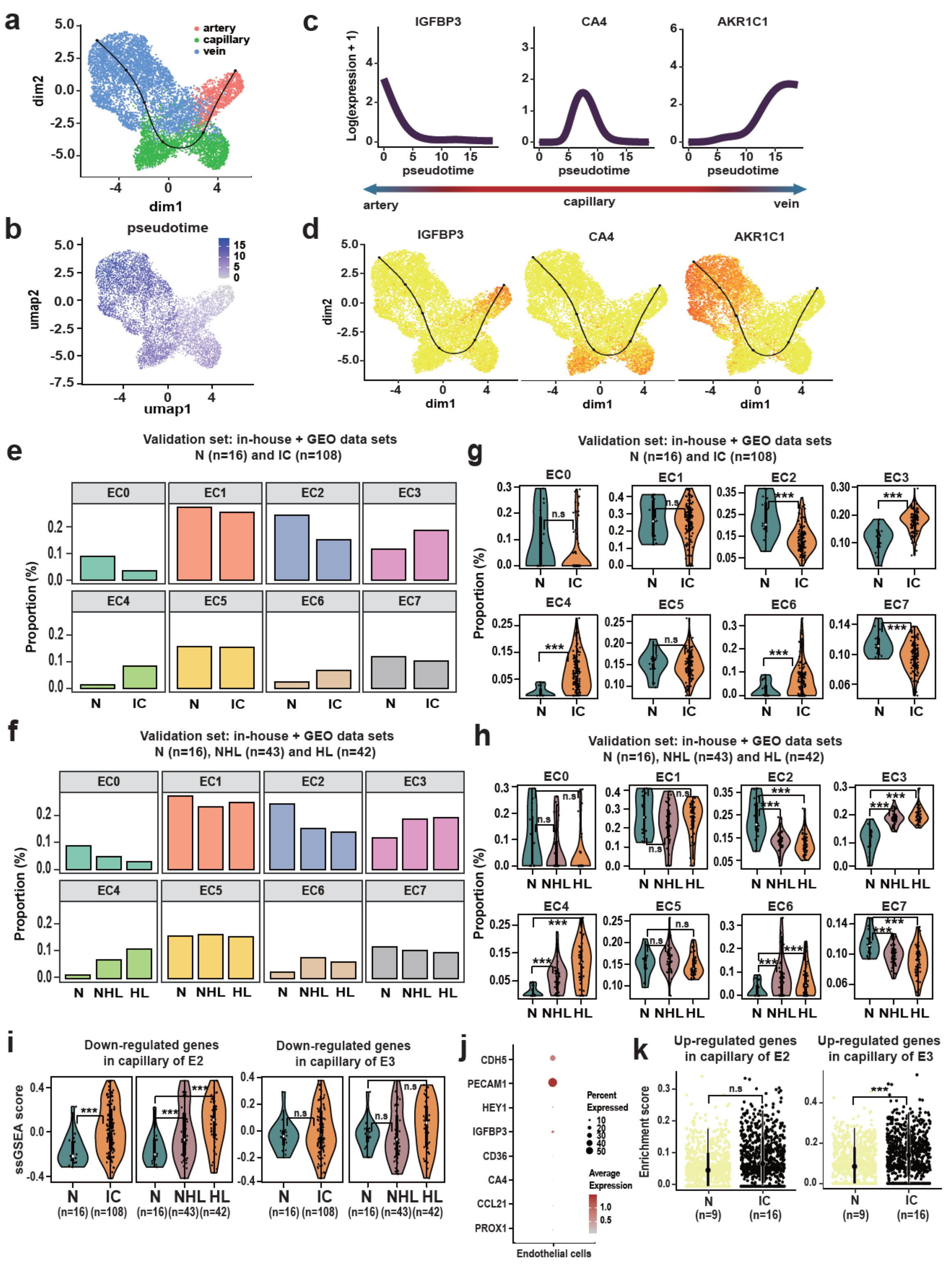

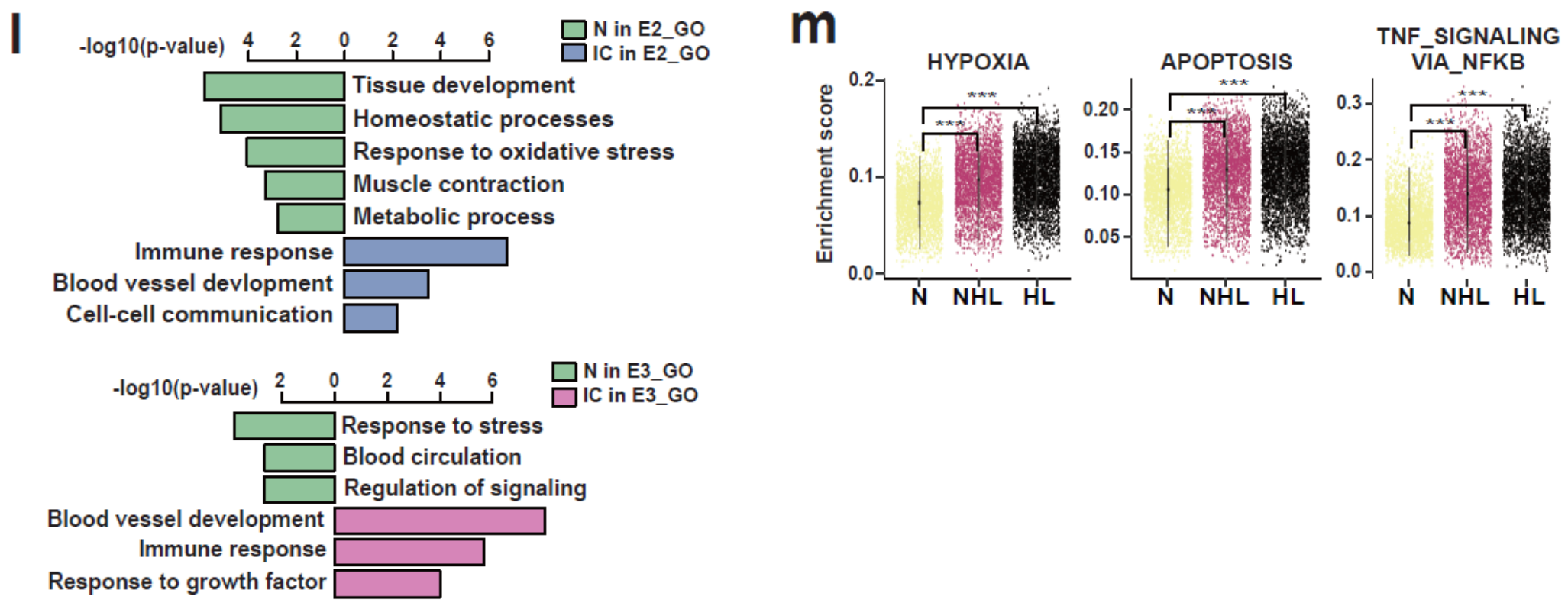

Supplementary Fig. 5. Pseudotime analysis of endothelial cells and external validation of sub-cluster dynamics. a. Pseudotime trajectory of endothelial cells generated using Slingshot. **b.** UMAP plot visualizing pseudotime trajectory across endothelial cells. **c.** Loess-smoothed expression patterns of endothelial subtype marker genes along the pseudotime trajectory. **d.** Expression dynamics of *IGFBP3*, *CA4*, and *AKR1C1* genes across pseudotime. **e, f.** Bar plots showing the proportions of endothelial sub-clusters across tissue types in the external validation datasets. **g, h.** Violin plots showing the proportions of endothelial sub-clusters in each patient across tissue types in the external validation datasets. **i.** Violin plots showing ssGSEA scores of downregulated genes in capillary E2 (left) and E3 (right) in external validation datasets. **j.** Average expression dot plot shows the endothelial lineage marker genes of endothelial cell type using public scRNA-seq datasets (the size of the circles indicates the percentage of cells expressing the gene, and the increasing color gradient from gray to pink to red corresponds to increasing expression values). **k.** Box plots illustrating ssGSEA enrichment scores of up-regulated genes in capillary E2 (left) and E3 (right) in public scRNA-seq datasets. **l.** GO analyses using differentially expressed genes in E2 and E3 cells between normal and IC tissues (including NHL and HL). **m.** Box plots showing ssGSEA scores for three selected Hallmark gene sets. (*p*-values for box plots were calculated using Student's *t*-test for two groups). NS, not significant; *$p < 0.05$; **$p < 0.01$; ***$p < 0.001$)

Supplementary Fig. 6

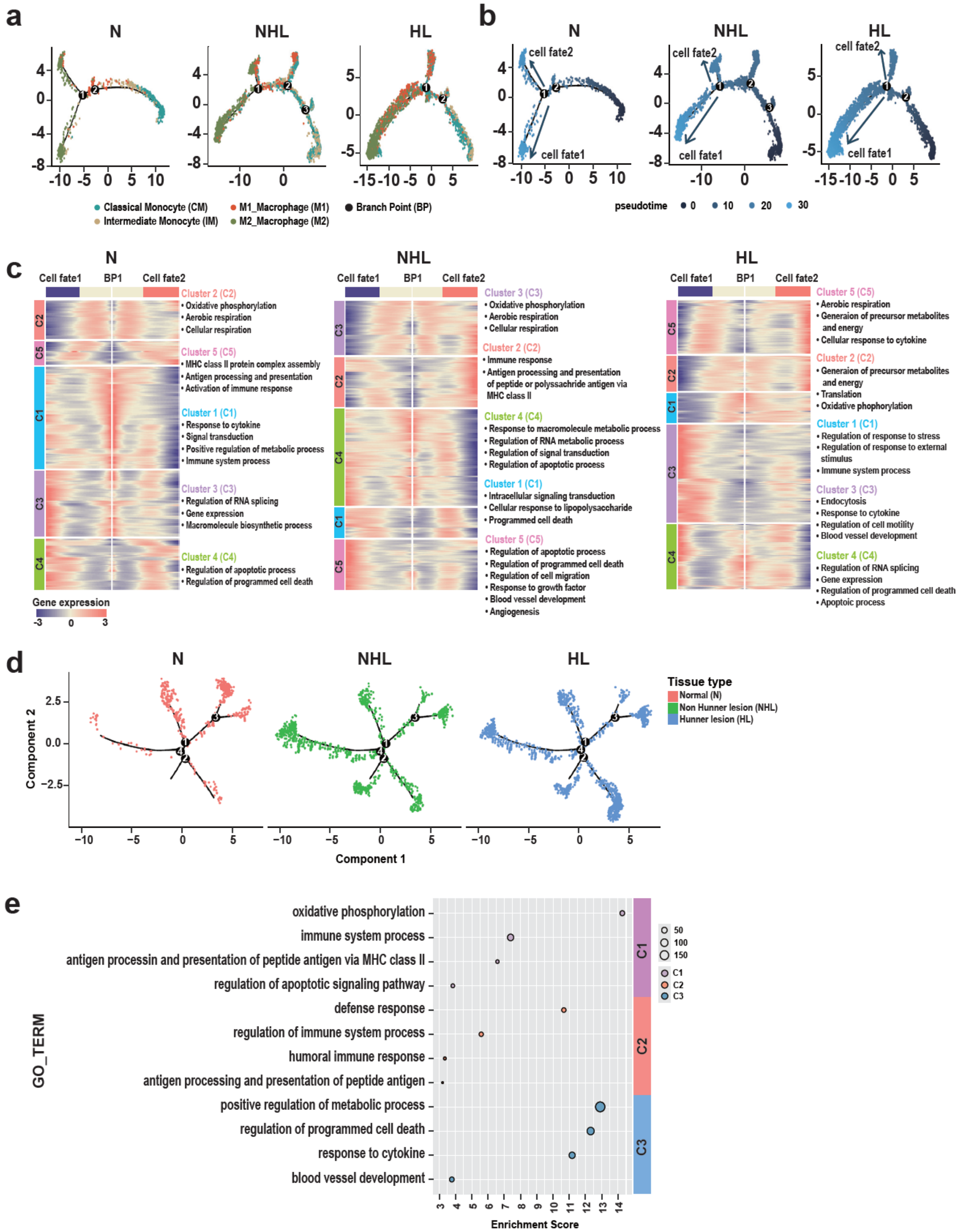

Supplementary Fig. 6. Pseudotime trajectory analysis of monocytes and macrophages across different tissue types. a, b. Pseudotime trajectory plot generated using Monocle2 for

monocytes and macrophages. **(a)** Trajectory plots stratified by tissue type. **(b)** Corresponding pseudotime plots. **c.** Heatmaps showing pseudotime-dependent gene expression clusters (C1–C5) at branch point 1 for each tissue type. GO analysis results for each cluster are shown in the right panel. **d.** Pseudotime trajectory plots of total M2 macrophages stratified by tissue type. **e.** GO enrichment analysis of gene modules (C1–C3) derived from the terminal branches (CF1 and CF2) originating at branch point 4 along the M2 pseudotime trajectory. (Dot plot showing enriched GO terms for each module, with dot size representing the number of genes and color indicating the corresponding gene module. The x-axis represents the enrichment score.)

Supplementary Fig. 7

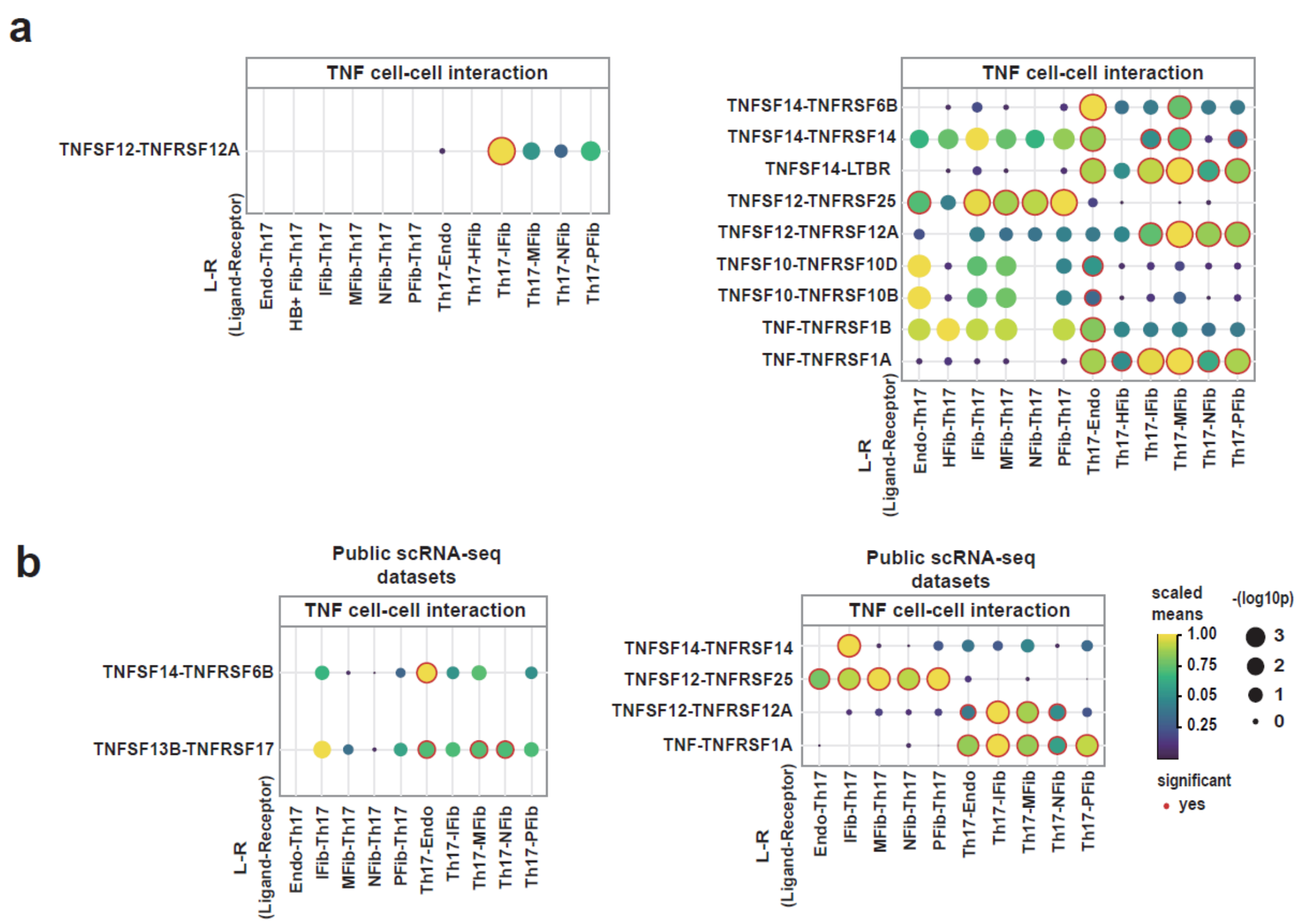

Supplementary Fig. 7. TNF-related cell–cell interaction analysis involving endothelial cells, Th17 cells, and fibroblasts. a. Ligand–receptor interaction analysis among Th17 cells, fibroblasts, and endothelial cells in our scRNA-seq dataset (normal, left; IC, right). **b.** Ligand–receptor interaction analysis among Th17 cells, fibroblasts and endothelial cells in public scRNA-seq datasets (normal, left; IC, right). (color intensity and circle size represent interaction significance based on $-\log_{10}(p)$; interactions labeled as significant indicate $p < 0.05$)

Supplementary Fig. 8

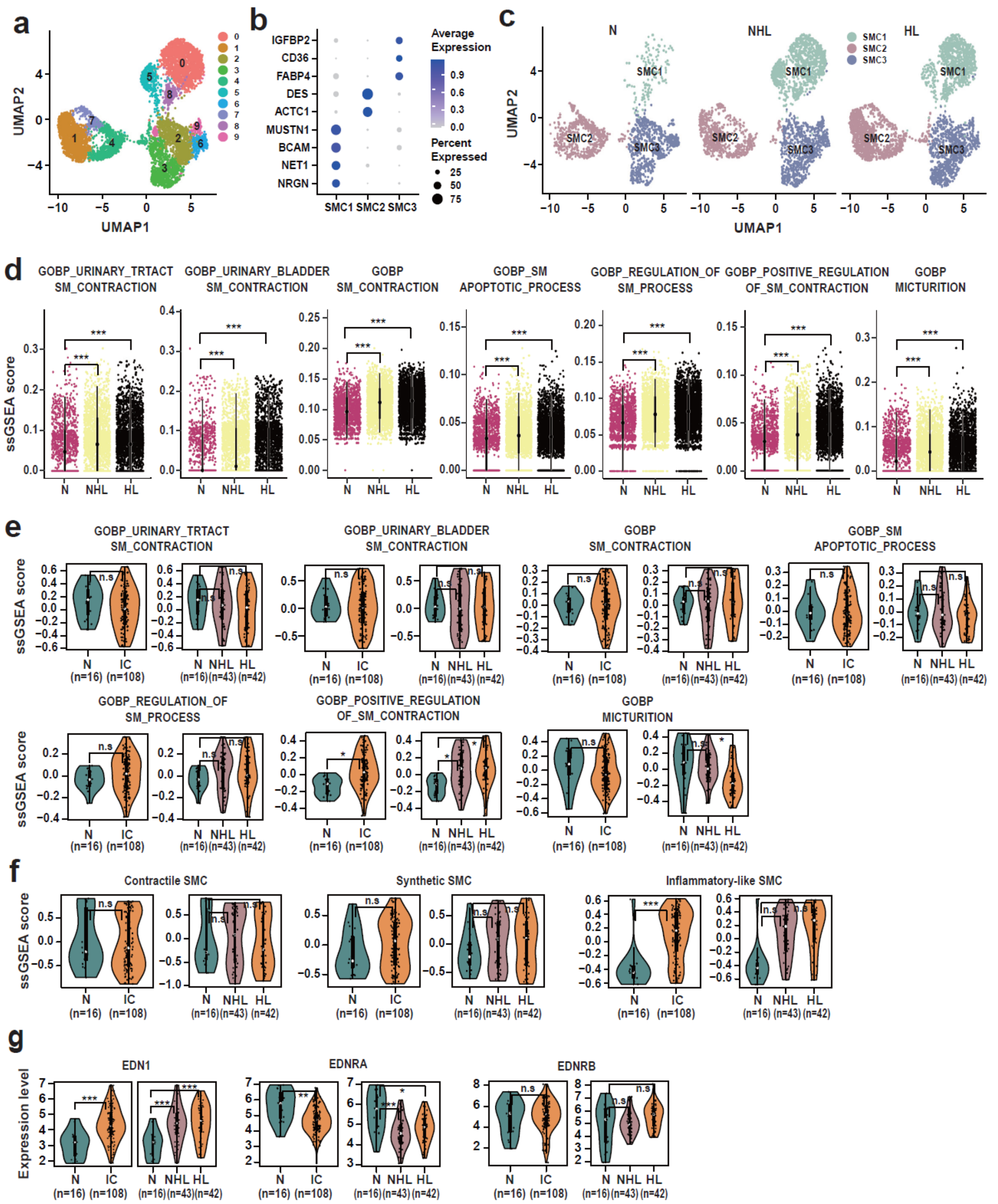

Supplementary Fig. 8. Characterization of SMCs and external validation of contraction-related gene sets. a. UMAP plot showing the 10 SMCs sub-clusters. **b.** Dot plots showing the expression levels of the top marker genes in each SMC subtype (dot size indicates the

percentage of cells expressing the gene, and the increasing color gradient from gray to purple to blue corresponds to increasing expression values). **c.** UMAP plots showing the three SMC subtypes stratified by tissue type. **d.** Box plots illustrating ssGSEA scores of contraction-related gene sets. **e.** Violin plots showing ssGSEA scores of contraction-related gene sets in the external validation datasets. **f.** Violin plots showing ssGSEA scores of SMC phenotype-related gene sets in the external validation datasets. **g.** Violin plots showing the expression levels of *EDN1*, *EDNRA*, and *EDNRB* in the external validation datasets. (Significance was determined using Student's *t*-test. NS, not significant; $^{*}p < 0.05$; $^{**}p < 0.01$; $^{***}p < 0.001$)

Supplementary Fig. 9

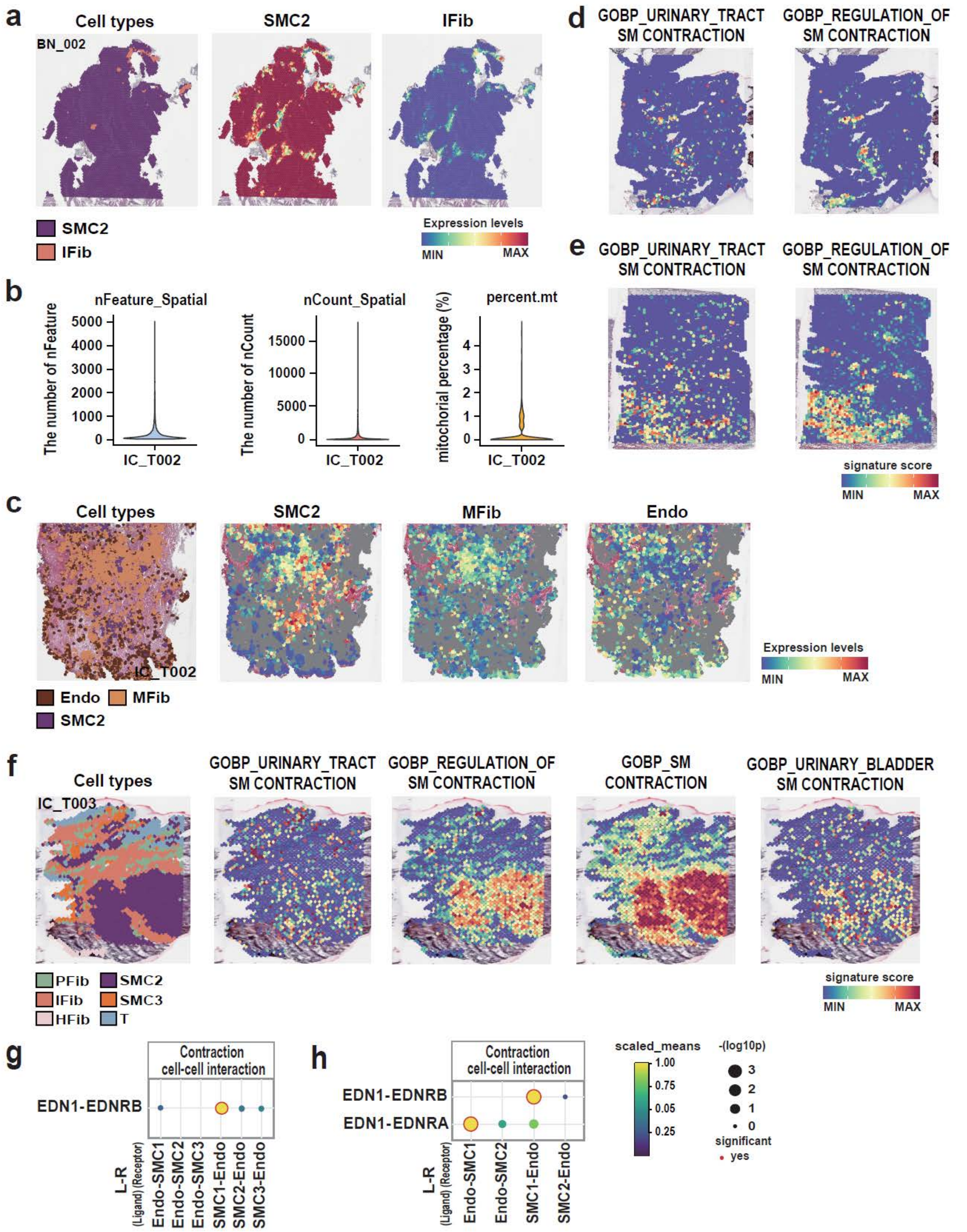

Supplementary Fig. 9. Spatial transcriptomic analysis of control and IC/BPS bladder tissue. a. Spatial transcriptomic (ST) map of the BN_002 sample and spatial plots showing the expression of the annotated cell type. **b.** Violin plots presenting quality control metrics of the

IC_T002 sample, including detected features (nFeature), number of transcripts (nCount), and percentage of mitochondrial genes. **c.** ST map of the IC_T002 sample and corresponding spatial plots displaying the expression of the annotated cell type. **d, e.** ST maps of control (top) and IC/BPS (bottom) tissues, colored by cell type based on CARD. Spatial plots showing contraction-related signature scores calculated using AddModuleScore in control (d) and IC/BPS (e) tissue samples. **f.** ST map of an additional IC/BPS sample (IC_T003), colored by cell type (CARD), along with the spatial distribution of contraction-related signature scores. **g, h.** Ligand–receptor interaction analysis between endothelial cells and SMCs in normal (BN_001) **(g)** and IC/BPS tissues (IC_T001) **(h)**. (color intensity and circle size represent interaction significance based on $-\log_{10}(p)$; interactions labeled as significant indicate $p < 0.05$)

Supplementary Fig. 10

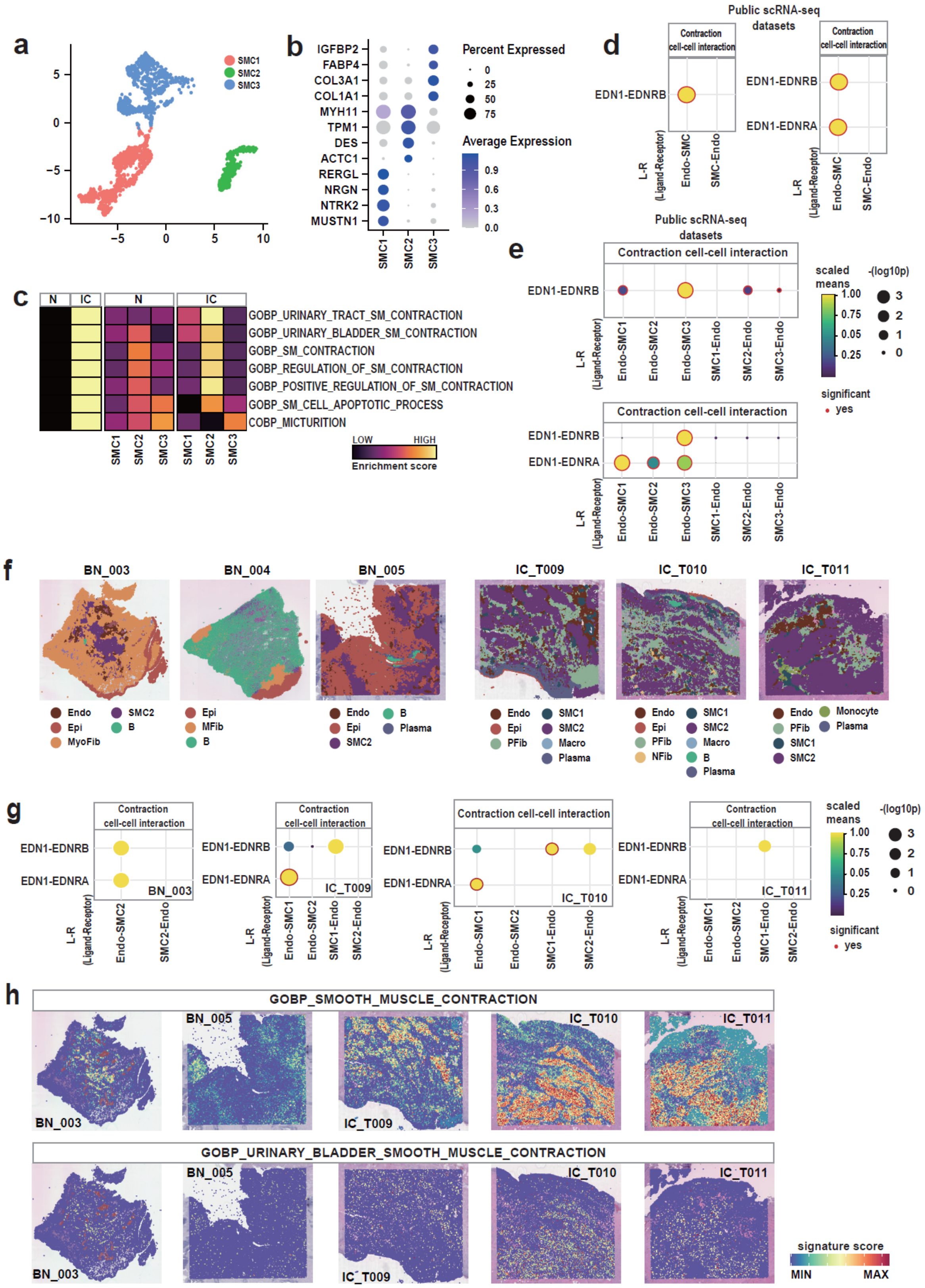

Supplementary Fig. 10. External validation of contraction-related features in normal and IC/BPS. a. UMAP plot displaying the annotation of SMC subtypes. **b.** Dot plots showing the expression levels of endothelial lineage marker genes using public scRNA-seq datasets (dot size indicates the percentage of cells expressing the gene and increasing color gradient from gray to purple to blue corresponds to an increasing expression value). **c.** Heatmap of GSEA scores of contraction-related gene sets across tissue types and SMC subtypes using public scRNA-seq datasets. **d.** Ligand–receptor interaction analysis between endothelial cells and SMCs in normal (left) and IC/BPS (right) tissues using public scRNA-seq datasets. **e.** Ligand–receptor interaction analysis between endothelial and SMC sub-clusters in normal (top) and IC/BPS (bottom) tissues using public scRNA-seq datasets. (color intensity and circle size represent interaction significance based on $-\log_{10}(p)$; interactions labeled as significant indicate $p < 0.05$). **f.** Visium HD maps of normal (left) and IC/BPS (right) tissues, colored by cell type based on ST deconvolution was conducted via RCTD, as provided by the spacexr R library. **g.** Ligand–receptor interaction analysis between endothelial cells and SMCs in normal and IC/BPS tissues using Visium HD data. (color intensity and circle size represent interaction significance based on $-\log_{10}(p)$; interactions labeled as significant indicate $p < 0.05$). **h.** Spatial maps showing contraction-related signature scores calculated using AddModuleScore in control and IC/BPS. (Epi, Epithelial cells; B, B cells; Endo, Endothelial cells; PFib, PDGFRA+ Fibroblasts, NFib, Neuron-like Fibroblasts; Macro, Macrophages; SMC, Smooth muscle cells)

Supplementary Fig. 11

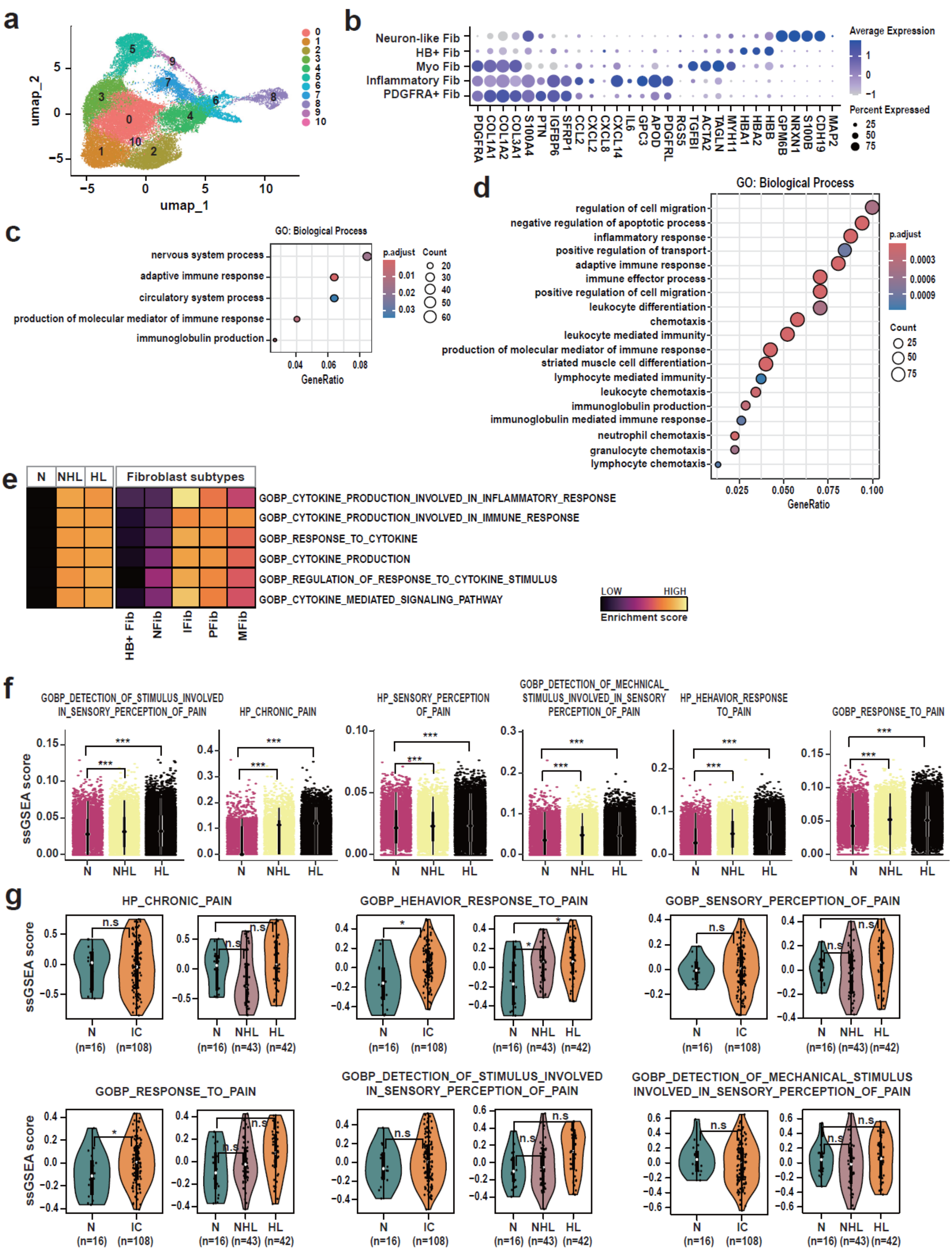

Supplementary Fig. 11. GO enrichment analysis of fibroblast subtypes and external validation of pain-related gene sets. a. UMAP plot displaying 11 transcriptionally distinct fibroblast sub-clusters. **b.** Dot plots showing the expression levels of representative marker genes across fibroblast subtypes. (dot size indicates the percentage of cells expressing the gene, and the increasing color gradient from gray to purple to blue corresponds to increasing expression value). **c, d.** Dot plots showing GO enrichment analysis of differentially expressed genes (DEGs) between IC/BPS and normal tissues in PDGFRA^{+} fibroblasts (PFib) **(c)** and inflammatory fibroblasts (IFib) **(d)**. The x-axis represents the gene ratio, and the y-axis indicates enriched GO terms. **e.** Heatmaps showing ssGSEA scores of cytokine-related gene sets across tissue types (left; N, NHLs, and HLs) and across fibroblast subtypes defined in IC/BPS tissues (right). **f.** Box plots showing ssGSEA scores of pain-related gene sets across tissue types. **g.** Violin plots showing ssGSEA scores of pain-related gene sets in the external validation datasets. (Significance was determined by Student's *t*-test. NS, not significant; *$p < 0.05$; **$p < 0.01$; ***$p < 0.001$)

Supplementary Fig. 12

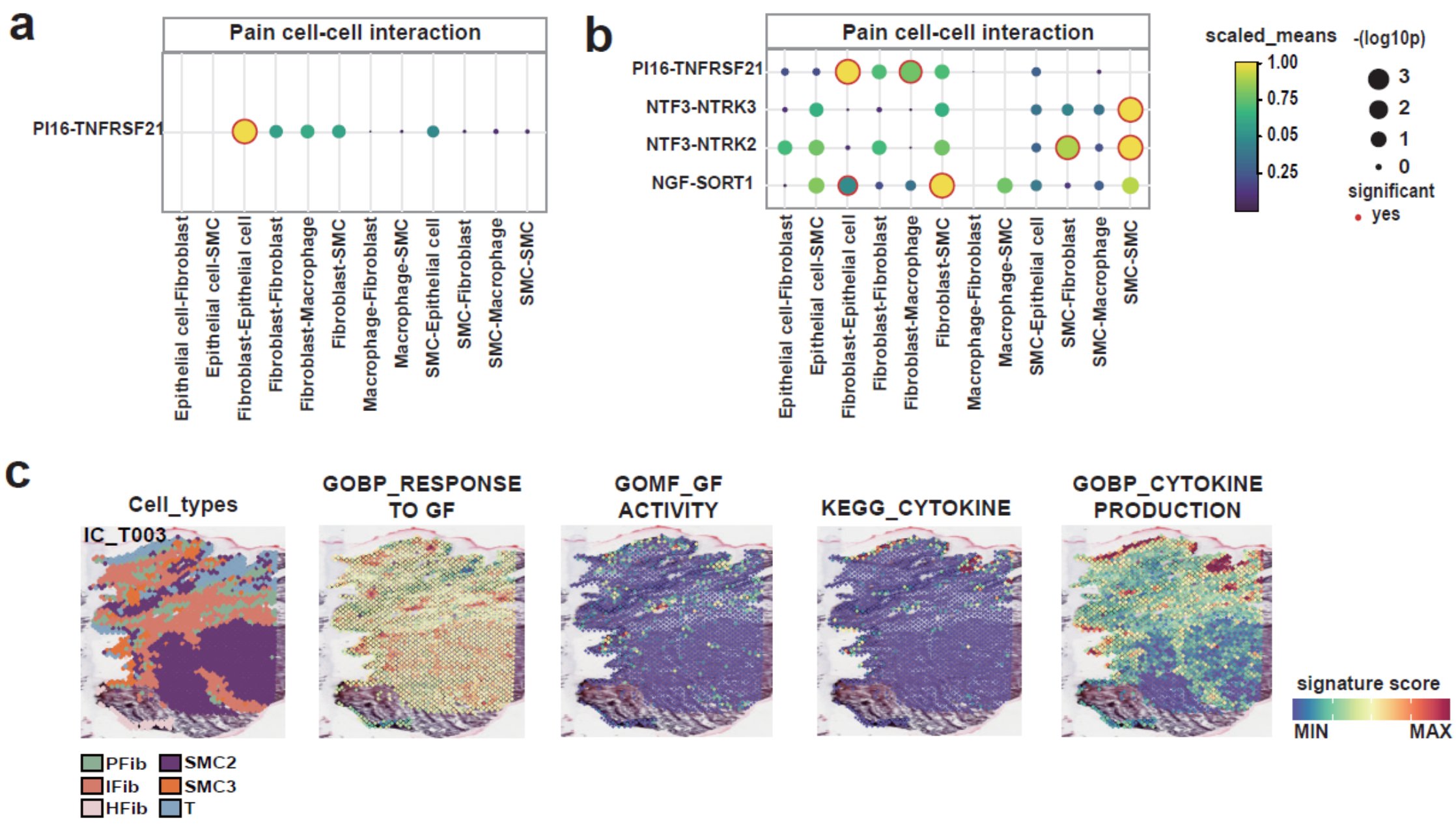

Supplementary Fig. 12. Ligand–receptor interaction analysis and spatial analysis of growth factor- and cytokine-related gene sets. a, b. Ligand–receptor pairs identified from the ST data of BN_001 **(a)** and IC_T001 **(b)** tissues. Circle size and color intensity represent statistical significance of interactions ($-\log_{10} p$); interactions marked as significant indicate $p < 0.05$. **c.** Spatial plots of the IC_T003 sample showing enrichment scores of cytokine- and growth factor(GF)-related gene sets calculated using AddModuleScore.

Supplementary Fig. 13

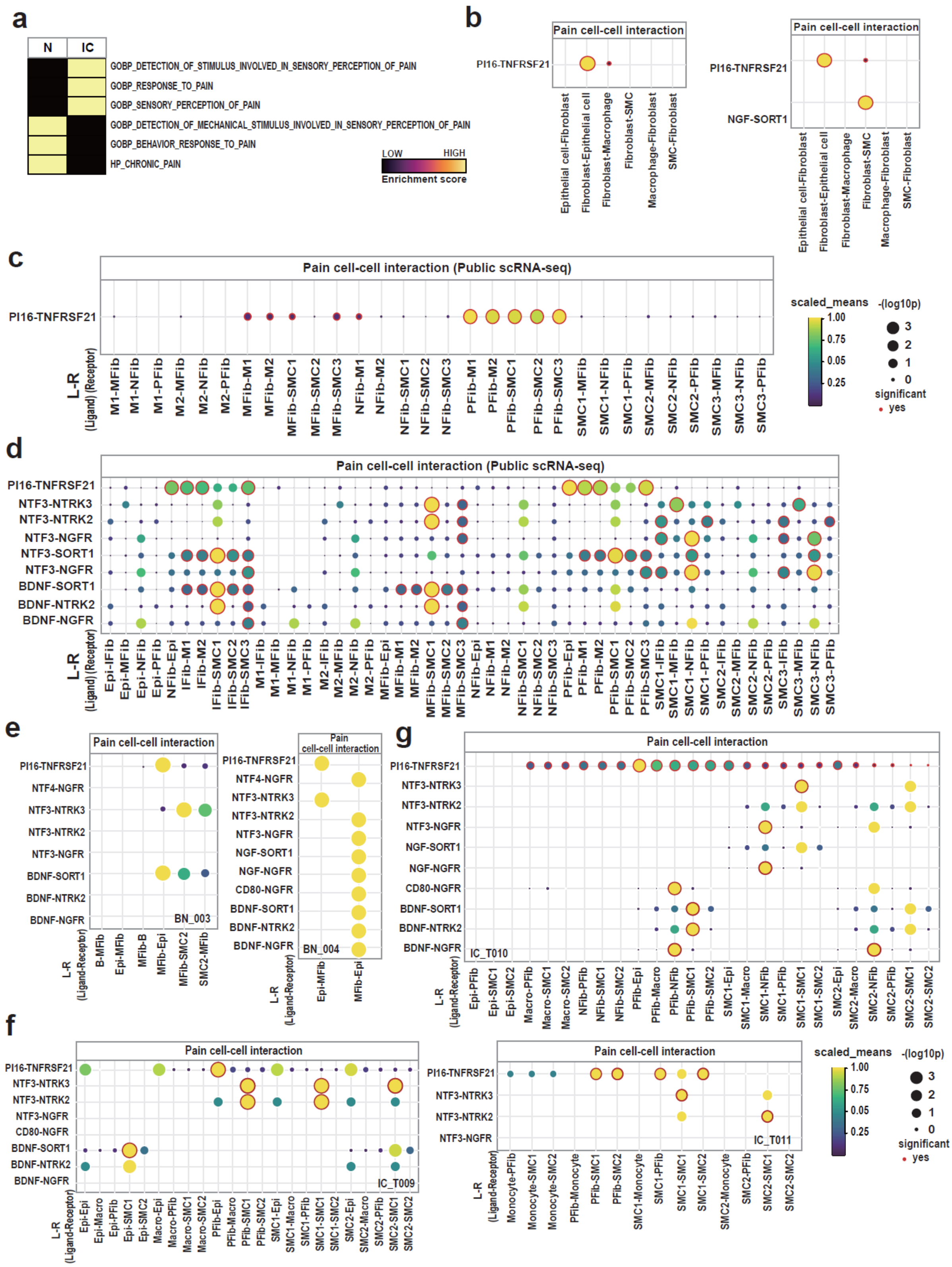

Supplementary Fig. 13. Ligand-receptor interaction analysis using public scRNA-seq and Visium HD datasets. a. Heatmap of GSEA scores of pain-related gene sets in fibroblast across tissue types of using public scRNA-seq datasets. **b.** Ligand–receptor interaction analysis from our scRNA-seq data in normal (left) and IC/BPS (right) tissues. **c, d.** Ligand–receptor interaction analysis inferred from public scRNA-seq datasets in normal **(c)** and IC/BPS tissues **(d)**. **e-g.** Ligand–receptor interaction analysis in normal **(e)** and IC/BPS tissues **(f, g)** using Visium HD data. (color intensity and circle size represent interaction significance based on $-\log_{10}(p)$; interactions labeled as significant indicate $p < 0.05$)

Supplementary Fig. 14

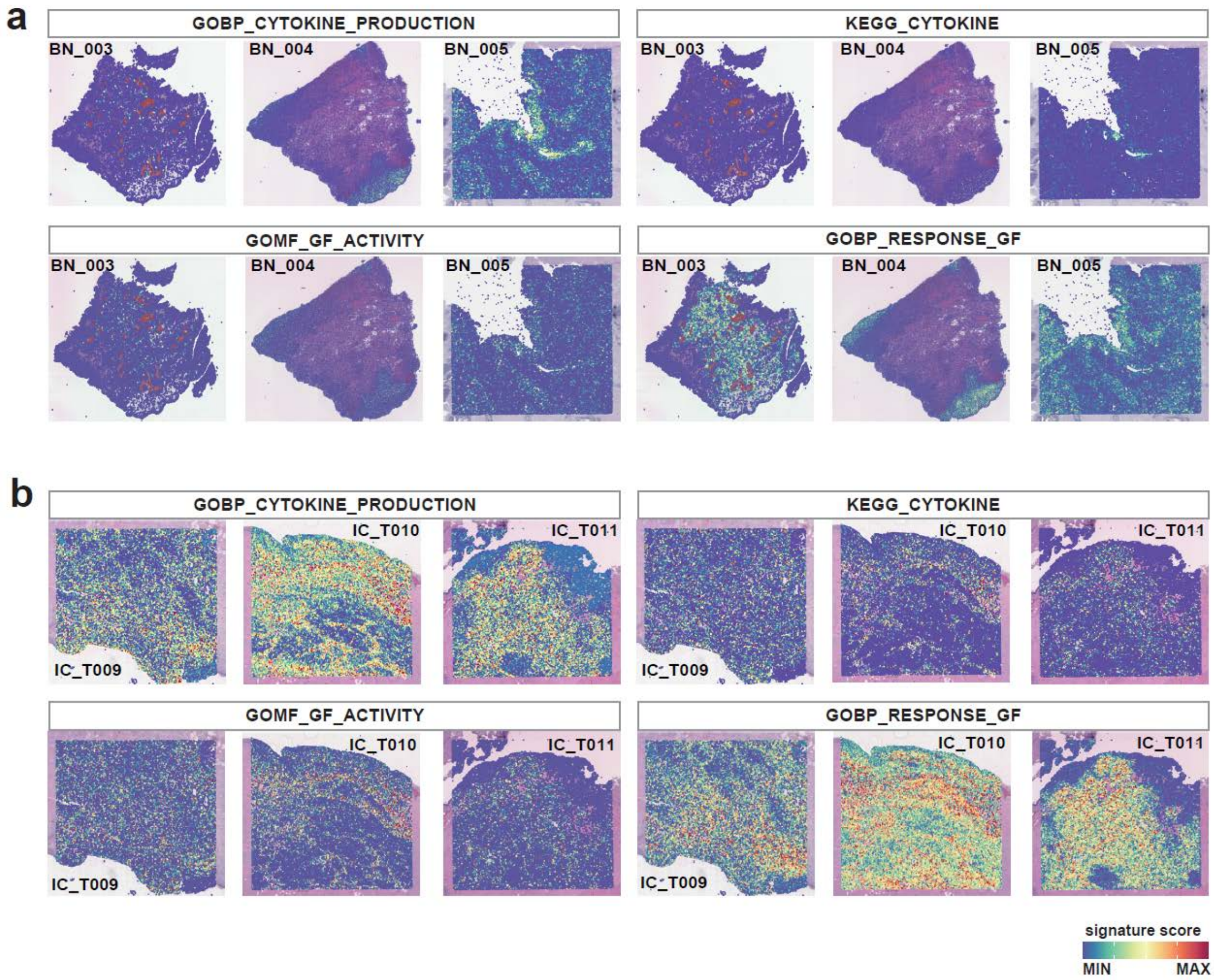

Supplementary Fig. 14. Validation of cytokine- and growth factor–associated spatial activation in IC/BPS using Visium HD. a, b. Spatial maps showing cytokine and growth factor (GF)-related signature scores calculated using AddModuleScore in control **(a)** and IC/BPS **(b)**. (BN, Bladder Normal; IC, Interstitial cystitis)

Supplementary Fig. 15

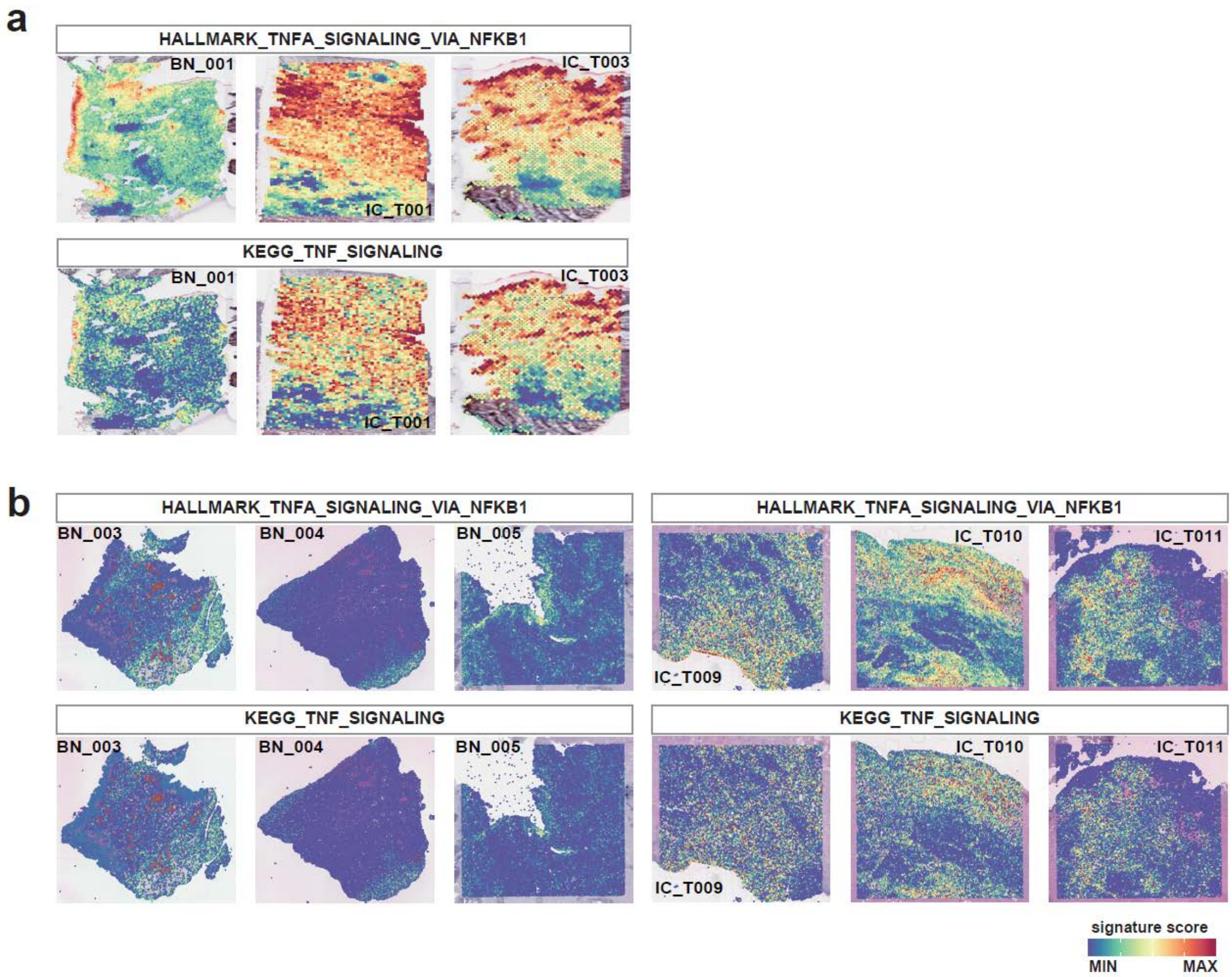

Supplementary Fig. 15. Validation of TNF–associated spatial activation in IC/BPS using Visium HD. a, b. Spatial maps showing TNF-related signature scores calculated using AddModuleScore in Visium **(a)** and Visium HD **(b)**. (BN, Bladder Normal; IC, Interstitial cystitis)

Supplementary Table 1. Clinical characteristics of patients with Hunner-type interstitial cystitis (n = 8).

Characteristic	Value
Age (y), median (range)	64.0 (55.5–72.6)
Sex, n (%)	
Male	5 (62.5)
Female	3 (37.5)
Number of endoscopic ablations, median (range)	3.0 (2–5)
Maximal cystometric capacity (mL), median (range)	195.0 (80.0–280.0)
Number of Urinary Frequency, median (range)	15.4 (9.3–33.3)
Mean bladder capacity (mL), median (range)	122.5 (57.0–178.0)
O'Leary–Sant Interstitial Cystitis questionnaire (IC-Q), median (range)	
Symptom index	15.5 (13.0–20.0)
Problem index	15.0 (11.0–16.0)
Pelvic pain and urgency/frequency (PUF) symptom scale, median (range)	
Symptom	17.0 (10.0–21.0)
Bothersome	8.5 (5.0–10.0)
Pain for VAS, median (range)	7.5 (4.0–10.0)

Supplementary Table 2. Clinical characteristics of each patient with IC/BPS (n = 8) and normal control (n = 2) samples.

	IC1	IC2	IC3	IC4	IC5	IC6	IC7	IC8	Control1	Control2
No. of prior endoscopic ablation	5	3	3	3	2	2	2	4	N/A	N/A
Age	82	70	65	47	63	54	78	58	66	65
Sex	F	F	M	M	M	F	M	M	F	M
Mean no. of Daytime frequency	10.3	33.3	17.7	11	28.3	9.3	19.3	13	5.7	5
Mean no. of Nocturia	2	9.3	2.3	1.3	5.7	1.3	4	4.5	0.7	0
Mean bladder capacity (mL)	155	57	145	178	73	116	92	129	169	184
Maximal cystometric capacity (mL)	200	80	190	280	90	250	125	210	400	300
ICSI	16	14	20	19	14	13	20	15	N/A	N/A
ICPI	15	12	16	16	12	11	16	15	N/A	N/A
PUF symptom	17	18	17	21	14	10	20	16	N/A	N/A
PUF bother	9	9	8	10	7	5	9	8	N/A	N/A
Pain VAS	8	8	7	4	5	8	10	7	N/A	N/A

ICSI, O'Leary–-Sant Interstitial Cystitis Symptom Index Questionnaire; ICPI, O'Leary–Sant Interstitial Cystitis Problem Index Questionnaire; N/A, not applicable; PUF, Pelvic pain and urgency/frequency; VAS, visual analog scale.
